# Impact of tumor microenvironment on efficacy of anti-CD19 CAR T cell therapy or chemotherapy and transplant in large B cell lymphoma

**DOI:** 10.1038/s41591-023-02754-1

**Published:** 2024-01-17

**Authors:** Frederick L. Locke, Simone Filosto, Justin Chou, Saran Vardhanabhuti, Regis Perbost, Peter Dreger, Brian T. Hill, Catherine Lee, Pier L. Zinzani, Nicolaus Kröger, Armando López-Guillermo, Hildegard Greinix, Wangshu Zhang, Gayatri Tiwari, Justin Budka, Francesco M. Marincola, Christina To, Mike Mattie, Marco Schupp, Paul Cheng, Adrian Bot, Rhine Shen, Davide Bedognetti, Harry Miao, Jérôme Galon

**Affiliations:** 1 Moffitt Cancer Center, Tampa, FL USA; 2 Kite, a Gilead Company, Santa Monica, CA USA; 3 Veracyte, Marseille, France; 4 Heidelberg University Hospital, Heidelberg, Germany; 5 Cleveland Clinic Foundation, Cleveland, OH USA; 6 Huntsman Cancer Institute, University of Utah, Salt Lake City, UT USA; 7 IRCCS Azienda Ospedaliero-Universitaria di Bologna Istituto di Ematologia Seràgnol and Dipartimento di Medicina Specialistica, Diagnostica e Sperimentale Università di Bologna, Bologna, Italy; 8 University Medical Center Hamburg, Hamburg, Germany; 9 Department of Hematology, Hospital Clinic, Barcelona, Spain; 10 Division of Hematology Medical University Graz, Graz, Austria; 11 INSERM, Sorbonne Université, Université Paris Cité, Centre de Recherche des Cordeliers, Equipe Labellisée Ligue Contre le Cancer, Laboratory of Integrative Cancer Immunology F-75006, Paris, France

**Keywords:** Predictive markers, B-cell lymphoma, Cancer immunotherapy, Chemotherapy

## Abstract

The phase 3 ZUMA-7 trial in second-line large B cell lymphoma demonstrated superiority of anti-CD19 CAR T cell therapy (axicabtagene ciloleucel (axi-cel)) over standard of care (SOC; salvage chemotherapy followed by hematopoietic transplantation) (NCT03391466). Here, we present a prespecified exploratory analysis examining the association between pretreatment tumor characteristics and the efficacy of axi-cel versus SOC. B cell gene expression signature (GES) and CD19 expression associated significantly with improved event-free survival for axi-cel (*P* = 0.0002 for B cell GES; *P* = 0.0165 for CD19 expression) but not SOC (*P* = 0.9374 for B cell GES; *P* = 0.5526 for CD19 expression). Axi-cel showed superior event-free survival over SOC irrespective of B cell GES and CD19 expression (*P* = 8.56 × 10^–9^ for B cell GES high; *P* = 0.0019 for B cell GES low; *P* = 3.85 × 10^–9^ for CD19 gene high; *P* = 0.0017 for CD19 gene low). Low CD19 expression in malignant cells correlated with a tumor GES consisting of immune-suppressive stromal and myeloid genes, highlighting the inter-relation between malignant cell features and immune contexture substantially impacting axi-cel outcomes. Tumor burden, lactate dehydrogenase and cell-of-origin impacted SOC more than axi-cel outcomes. T cell activation and B cell GES, which are associated with improved axi-cel outcome, decreased with increasing lines of therapy. These data highlight differences in resistance mechanisms to axi-cel and SOC and support earlier intervention with axi-cel.

## Abstract (web summary)

Analysis of the pivotal phase 3 ZUMA-7 trial identifies tumor gene expression signatures that are uniquely predictive of anti-CD19 CAR T cell response and event-free survival in second-line treatment for patients with relapsed or refractory large B cell lymphoma.

## Main

Axi-cel is an autologous anti-CD19 chimeric antigen receptor (CAR) T cell therapy initially approved for the treatment of relapsed/refractory large B cell lymphoma (LBCL) in adults after at least two lines of systemic therapy. ZUMA-7 (NCT03391466) was a randomized, international, multicenter phase 3 study of axi-cel versus SOC (defined as two or three cycles of protocol-defined, investigator-selected, platinum-based chemotherapy with intention to subsequently undergo high-dose chemotherapy with autologous stem cell transplantation (HDT-ASCT) for chemosensitive patients^1^) as second-line treatment in patients with LBCL who were refractory to, or had relapsed no more than 12 months after, first-line chemoimmunotherapy. Axi-cel was superior to SOC, with significant improvement in efficacy, and displayed a manageable safety profile^1^. In the primary event-free survival (EFS) analysis, the EFS hazard ratio was 0.398 (*P* < 0.0001; median EFS of 8.3 versus 2.0 months and estimated 24-month EFS rates of 40.5% versus 16.3% in the axi-cel versus SOC arms, respectively). Despite these striking results, a substantial number of patients presented primary (no response) or secondary (relapse after initial response) resistance to CAR T cell therapy, warranting further investigation into potential biomarkers associated with treatment resistance.

In LBCL, among clinical and real-world evidence in the chemoimmunotherapy era, known prognostic factors include high tumor burden, elevated lactate dehydrogenase (LDH), activated B cell (ABC)-like molecular subgroup, age and systemic inflammatory markers like interleukin-6 and C-reactive protein^2–9^. In the cellular therapy era, as shown in ZUMA-1 (third-line or higher LBCL), tumor burden and LDH associated negatively with efficacy to CAR T cell therapy^6^. Additionally, quality and quantity of pretreatment tumor infiltration of T cells, as characterized by ImmunoSign 21 (IS21; T cell gene expression signature (GES)) and by Immunoscore (immunohistochemistry (IHC) with CD3 and CD8 cells), associated positively with outcomes to CAR T cell therapy^10^. Translational data from patients treated with CAR T cell therapy in the real world further highlight the impact of tumor-associated chronic inflammation, checkpoint ligand upregulation, myeloid cell suppression of CAR T cell function^11^ and an association between patterns of tumor genomic complexity and CAR T cell outcomes^12^. Nonetheless, predictive biomarkers for CAR T cell intervention across lines of therapy are not well established, and the associations between tumor gene expression profiles and responses have not been investigated exhaustively^2,13^. The importance of the immune contexture within the tumor for CAR T cell therapy remains elusive^10,14–18^.

Here, we performed analyses of pretreatment tumor characteristics in ZUMA-7 to discover tumor-specific features predictive of axi-cel or SOC efficacy.

## Results

### B cell GES associates with EFS and duration of response post axi-cel

**Fig. 1:**
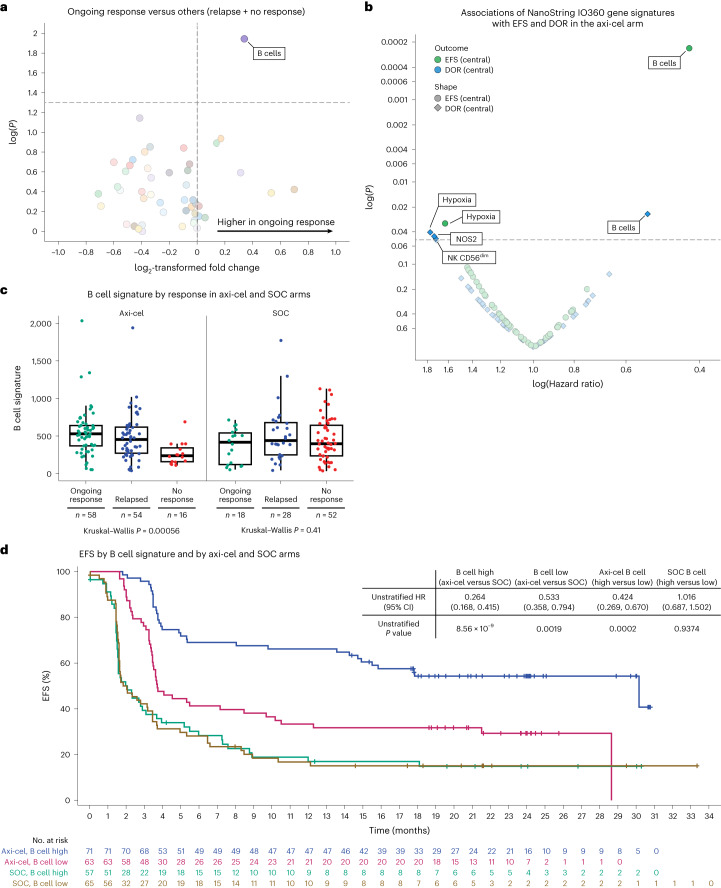
High B cell gene signature was associated with improved EFS and higher probability of durable response after axi-cel. **a**, Association of ongoing response with IO360 signatures as a volcano plot. The plot presents descriptive *P* value and fold change of IO360 signatures in ongoing response versus others (response followed by progressive diseases and no response) in the axi-cel arm. The fold change is presented as log_2_((group one)/(group two)). Statistical analyses were conducted using Kruskal–Wallis test (numerical versus categorical). **b**, NanoString IO360 GES associated with DOR (blue data points) and EFS (green data points) in the axi-cel arm. Two-sided *P* values were calculated via a Cox proportional hazards model. **c**, B cell gene signature by response (where *n* reflects the number of independent patients with each response type) in the axi-cel (left; *n* = 58, ongoing response; *n* = 54, relapsed; *n* = 16, no response) and SOC (right; *n* = 18, ongoing response; *n* = 28, relapsed; *n* = 52, no response) arms. The box plots show quartile 1 (Q1), median and Q3, and the lower and upper whiskers show Q1 – 1.5 × interquartile range (IQR) and Q3 + 1.5 × IQR, respectively. **d**, Kaplan–Meier estimate of EFS by B cell gene signature and treatment arm (axi-cel versus SOC). Patients who did not meet the criteria for an event had their data censored (tick marks). Unstratified Cox proportional hazards *P* values (two-sided) are presented. CI, confidence interval.

**Extended Data Fig. 1:**
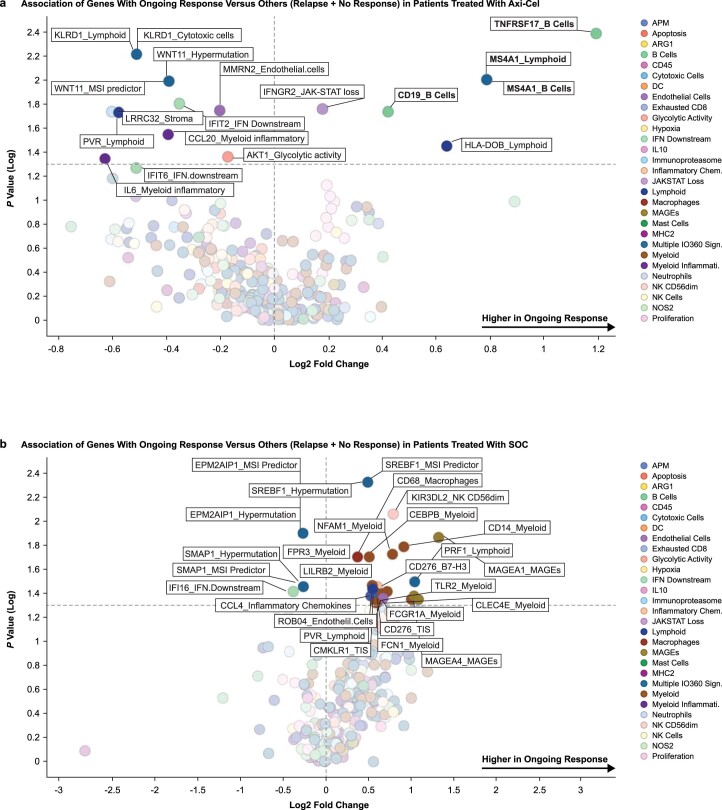
Elevation of select B-cell genes was associated with ongoing response. Panel a shows the association of ongoing response with genes included in Nanostring IO360™ GES as a volcano plot in axi-cel treated patients. The corresponding Nanostring IO360™ GES for each gene is appended to the gene name in the label. The plot presents the descriptive two-sided *P* value and fold change of each gene in ongoing responders versus others (response followed by progressive disease and no response) in the axi-cel arm. The fold change is presented as Log2([median Ongoing]/[median Others]). Statistical analyses were conducted using the Wilcoxon rank sum test (numerical vs categorical). Panel b shows the association of ongoing response with genes included in Nanostring IO360™ GES as a volcano plot in SOC-treated patients. The corresponding Nanostring IO360™ GES for each gene is appended to the gene name in the label. The plot presents the descriptive two-sided *P* value and fold change of each gene in ongoing responders versus others (response followed by progressive disease and no response) in the SOC arm. The fold change is presented as Log2([median Ongoing]/[median Others]). Statistical analyses were conducted using the Wilcoxon rank sum test (numerical vs categorical). axi-cel, axicabtagene ciloleucel; SOC, standard of care.

To identify markers associated with outcome in LBCL, an exploratory prespecified gene expression analysis of pretreatment tumor biopsies (based on available samples collected either at initial diagnosis or before lymphodepleting chemotherapy) was performed, leveraging the NanoString PanCancer IO360 Panel to evaluate predefined GES (Supplementary Table 1). The association of signatures with outcomes was analyzed (Fig. 1). In second-line axi-cel-treated patients, the B cell signature (IO360) was the only predefined signature associated *P* = 0.01) with higher probability of ongoing response (versus progression after response and no response; Fig. 1a,c), and improved EFS (*P* = 0.00024) and duration of response (DOR; *P* = 0.024; patients with high signature value (>median) versus those with low value (≤median); Fig. 1b,d). The prespecified B cell lineage signature included *BLK*, *CD19*, *MS4A1*, *TNFRSF17*, *FCRL2*, *FAM30A*, *PNOC*, *SPIB* and *TCL1A* genes. Of those genes, expression of *CD19*, *MS4A1* and *TNFRSF17* was elevated significantly among axi-cel (*P* = 0.0182, *P* = 0.0098 and *P* = 0.0040, respectively) but not SOC patients in ongoing response, with fold increases of 22%, 40% and 69%, respectively (Extended Data Fig. 1). Conversely, expression of hypoxia was associated significantly with shorter EFS (*P* = 0.032) and expression of hypoxia, nitric oxide synthase 2 (*NOS2*) and the natural killer (NK) CD56^dim^ NanoString signature were associated significantly with shorter DOR (*P* = 0.04, *P* = 0.045 and *P* = 0.048, respectively; Fig. 1b).

**Fig. 2:**
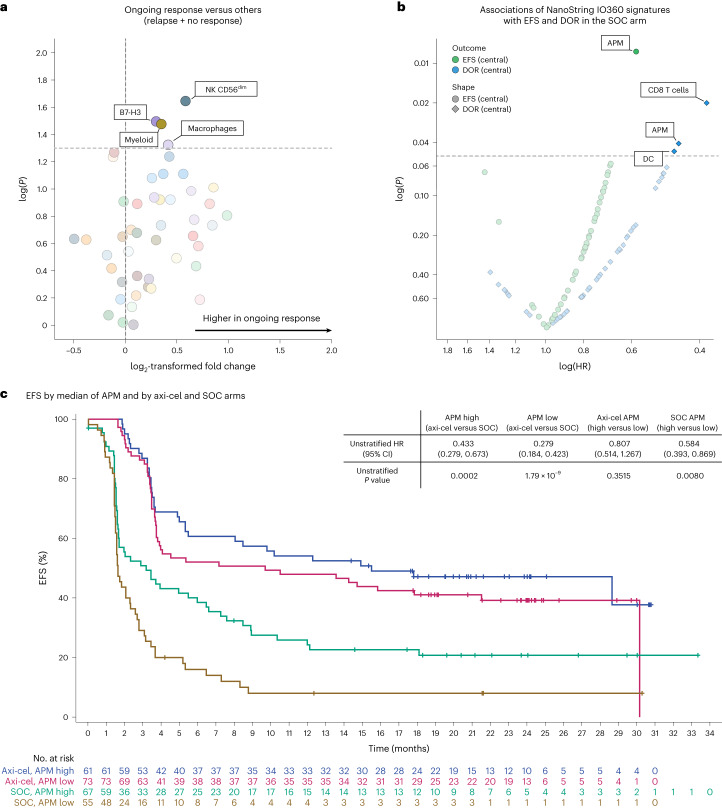
Association of NanoString IO360 signatures with ongoing response, EFS and DOR in the SOC arm. **a**, Association of ongoing response with IO360 signatures as a volcano plot. The plot presents descriptive *P* value and fold change of IO360 signatures in ongoing response versus others (response followed by progressive disease and no response) in the SOC arm. The fold change is presented as log_2_((group one)/(group two)). Statistical analyses were conducted using Kruskal–Wallis test (numerical versus categorical). **b**, NanoString IO360 GES associated with DOR (blue data points) and EFS (green data points) in the SOC arm. Two-sided *P* values were calculated via Cox proportional hazards model. **c**, Kaplan–Meier estimate of EFS by median APM and treatment arm (axi-cel versus SOC). Patients who did not meet the criteria for an event had their data censored (tick marks). Unstratified Cox proportional hazards *P* values (two-sided) are presented. DC, dendritic cells.

In the SOC arm, the B cell signature was not associated with efficacy, and few NanoString signatures associated with efficacy endpoints (Fig. 2a,b). Immune GES for macrophages, myeloid, antigen presentation machinery (APM), NK or CD8 T cells were associated with either ongoing response, EFS and/or DOR (none of the signatures associated consistently with all three efficacy metrics), suggesting that enrichment of select tumor immune infiltrates might be a factor supporting SOC responses. Nonetheless, axi-cel EFS was improved versus SOC for all subgroups, including high APM (*P* = 0.0002 > median; Fig. 2c).

### GES clusters revealed distinct tumor microenvironment immune contextures

**Fig. 3:**
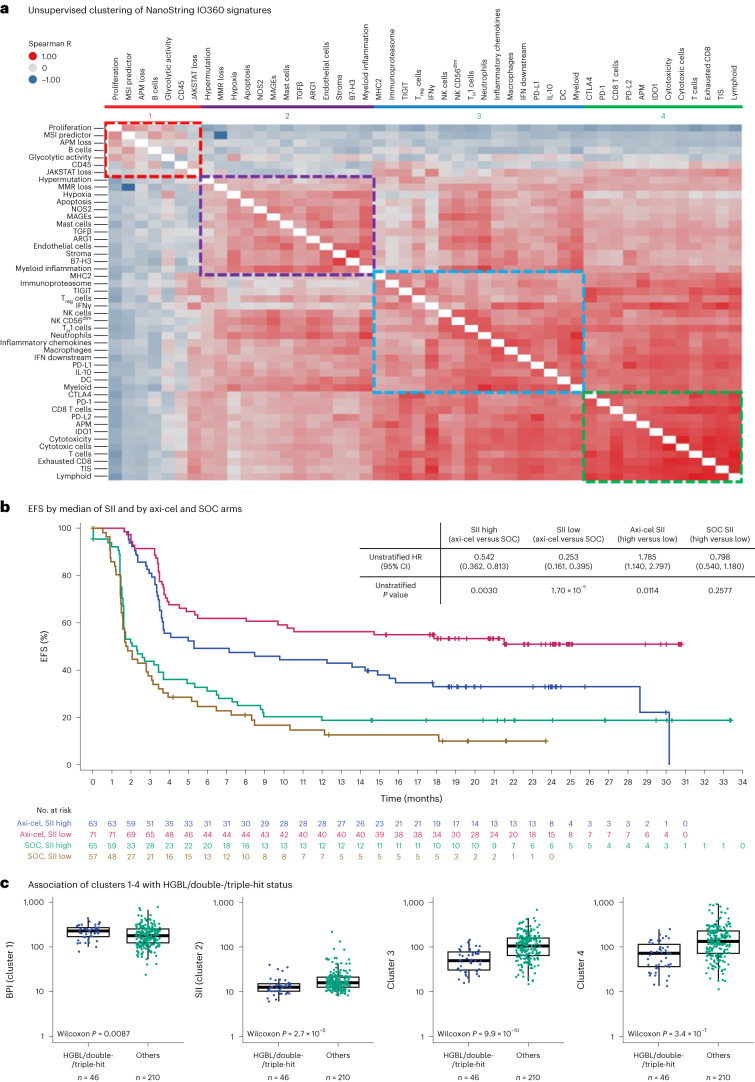
NanoString IO360 signature clustering and their association with EFS and HGBL status in the axi-cel arm. **a**, Unsupervised clustering of NanoString IO360 GES by Spearman rank-order correlation. Group 1 (red) represents BPI, which includes relatively low non B cell infiltration genes. Group 2 (purple) represents SII, which includes stromal and immune-suppressive genes. Group 3 (blue) represents mostly NK and myeloid cells (immune infiltration genes). Group 4 (green) represents mostly T lymphocytes (immune infiltration genes). Indices were calculated by the room mean square method. **b**, Kaplan–Meier estimate of EFS by SII and treatment arm (axi-cel versus SOC). Patients who did not meet the criteria for an event had their data censored (tick marks). Unstratified Cox proportional hazards *P* values (two-sided) are presented. **c** Association between HGBL/double-/triple-hit status (*n* = 46) versus other disease type (*n* = 210), where *n* reflects the number of independent patients with each disease type, with the four clusters. HGBL status correlated positively with BPI. The box plots show Q1, median and Q3, and the lower and upper whiskers show Q1 – 1.5 × IQR and Q3 + 1.5 × IQR, respectively. Two-sided *P* values were calculated per Wilcoxon test and are reported. IFN, interferon; IL-10, interleukin-10; JAKSTAT, Janus kinase signal transducer and activator of transcription; MAGE, melanoma antigen gene; MHC, major histocompatibility complex; MMR, mismatch repair; MSI, microsatellite instability; PD-1, programmed cell death protein 1; PD-L1, programmed death-ligand 1; T_H_1, T helper type 1; TIS, tumor inflammation signature; T_reg_ cell, regulatory T cell.

Based on unsupervised clustering analyses of NanoString IO360 GES, four main clusters were identified, underlying different tumor microenvironment (TME) immune contextures (Fig. 3a and Supplementary Table 1). The first cluster, herein referred to as the B cell lineage and proliferation index (BPI), included signatures like B cell, proliferation, APM loss and glycolytic activity. Signatures from BPI presented the highest hierarchical separation from the other three main clusters, suggesting a relatively simpler TME with abundant and highly proliferative cancer cells and lower immune cell infiltration versus the other clusters. A second cluster, termed the stromal and immunosuppressive index (SII), featured gene sets inclusive of stroma, myeloid and endothelial cells, NOS2, transforming growth factor-β (TGFβ), B7-H3, arginase1 (ARG1) and hypoxia. In this cluster, hypoxia and NOS2 signatures (IO360) were associated negatively with EFS and/or DOR following axi-cel treatment (Fig. 1b). A third cluster was enriched for signatures of NK cells, macrophages and antigen-presenting cells. A fourth cluster consisted primarily of T cell infiltration features. The third and fourth clusters showed a relatively close hierarchical correlation, perhaps jointly representing tumors that are more complex and immune infiltrated.

In addition, Supplementary Fig. 1 reports clustering of the individual genes from which the IO360 signatures are derived, presenting the directionality of the association of each gene for the IO360 signatures and the possibility to uncover further subclusters.

### SII and BPI associated with EFS in the axi-cel arm

**Extended Data Fig. 2:**
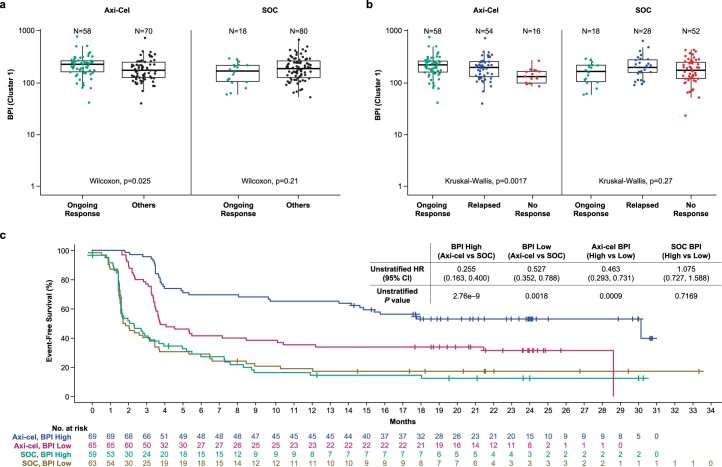
BPI correlated with responses to axi-cel. Panels **a** and **b** show association between cluster 1 (BPI) and ongoing response in the axi-cel (left) and SOC (right) arm. Panel a includes ongoing responders (n = 58, axi-cel; n = 18, SOC) versus others (combined progression after response and no response; n = 70, axi-cel; n = 80, SOC), whereas Panel b includes ongoing responders (n = 58, axi-cel; n = 18, SOC) versus progression after response (n = 54, axi-cel; n = 28, SOC) versus no response (n = 16, axi-cel; n = 52, SOC). For panels a-b, box plots show Q1, median, and Q3, and the lower and upper whiskers show Q1-1.5(IR) and Q3 + 1.5(IR), respectively; n values reflect the number of independent patients in each respective group. For panels a-b, two-sided *P* values were calculated per Wilcoxon test and Kruskal-Wallis test, respectively, and are reported. Panel **c** shows the Kaplan-Meier estimate of EFS by BPI and treatment arm (axi-cel vs SOC). Patients who did not meet the criteria for an event had their data censored (tick marks). Unstratified Cox proportional hazards *P* values (two-sided) are presented. Axi-cel, axicabtagene ciloleucel; BPI, B-Cell Lineage and Proliferation Index; CI, confidence interval; EFS, event-free survival; HR, hazard ratio; IR, interquartile; Q, quartile; SOC, standard of care.

**Extended Data Fig. 3:**
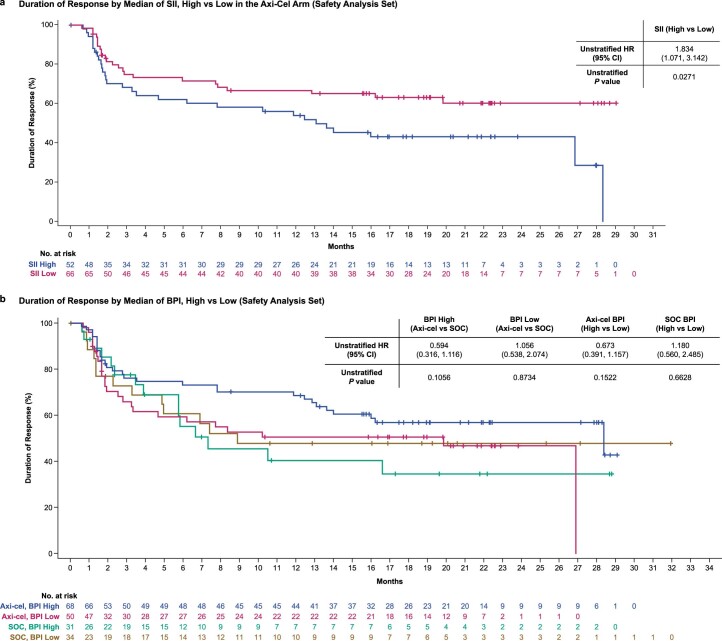
SII and BPI clusters negatively and positively, respectively, associated with DOR following axi-cel treatment. **a**,**b**, Kaplan-Meier estimate of DOR by SII (**a**) and by BPI (**b**) in the axi-cel treatment arm (SII High versus SII Low; BPI High vs BPI Low). Patients who did not meet the criteria for an event had their data censored (tick marks). Unstratified Cox proportional hazards *P* value (two-sided) is presented. Axi-cel, axicabtagene ciloleucel; BPI, B-cell lineage and proliferation index; SII, stromal and immunosuppressive index; axi-cel, axicabtagene ciloleucel.

**Extended Data Fig. 4:**
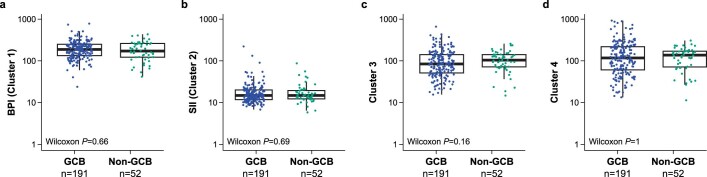
Gene expression clusters did not associate with cell of origin by molecular subgroup. The relation between cell of origin (GCB [n = 191] versus non-GCB [n = 52]) with the 4 clusters is depicted. Due to the limited number of unclassified subtypes in ZUMA-7, ABC (activated B-cell) subgroup and unclassified were grouped together as non–GCB-like. Box plots show Q1, median, and Q3, and the lower and upper whiskers show Q1-1.5(IR) and Q3 + 1.5(IR), respectively; n values reflect the number of independent patients in each respective group. Two-sided *P* values were calculated per Wilcoxon test and are reported. BPI, B-Cell Lineage and Proliferation Index; GCB, germinal center B-cell subgroup; IR, interquartile; Q, quartile; SII, Stromal and Immunosuppressive Index.

By root mean square, the signatures from each of the four clusters of predefined NanoString signatures (Fig. 3a) were combined to create indices for further analysis. BPI (cluster 1) and SII (cluster 2) were associated positively and negatively, respectively, with EFS and DOR in the axi-cel arm (EFS: *P* = 0.0009 for BPI and *P* = 0.0114 for SII; DOR: *P* = 0.1522 for BPI and *P* = 0.0271 for SII; Fig. 3b and Extended Data Figs. 2 and 3). The third and fourth clusters were not associated with EFS and/or DOR following axi-cel (Supplementary Fig. 2). Notably, none of these biomarkers associated with grade ≥3 cytokine release syndrome or neurologic events (Supplementary Tables 2 and 3). None of the four clusters were associated significantly with outcome in the SOC arm (BPI shown in Extended Data Fig. 2c). None of the four clusters associated with cell of origin (Extended Data Fig. 4). Notably, BPI associated positively with high-grade B cell lymphoma (HGBL) and double-/triple-hit disease (Fig. 3c). Median EFS in the HGBL subgroup following axi-cel treatment was 21.5 months (95% CI, 3.7–not evaluable; unstratified hazard ratio (HR) (axi-cel over SOC) = 0.318). EFS of the HGBL subtype was not significantly different from that of the non-HGBL subtype (DLBCL + others) in the axi-cel arm, albeit a directionally favorable HR was seen (unstratified HR (HGBL over non-HGBL) = 0.692; 95% CI, 0.384–1.245). The SOC arm showed an opposite trend (unstratified HR (HGBL over non-HGBL) = 1.17; 95% CI, 0.723–1.892).

### CD19 expression had differential impact on efficacy

**Fig. 4:**
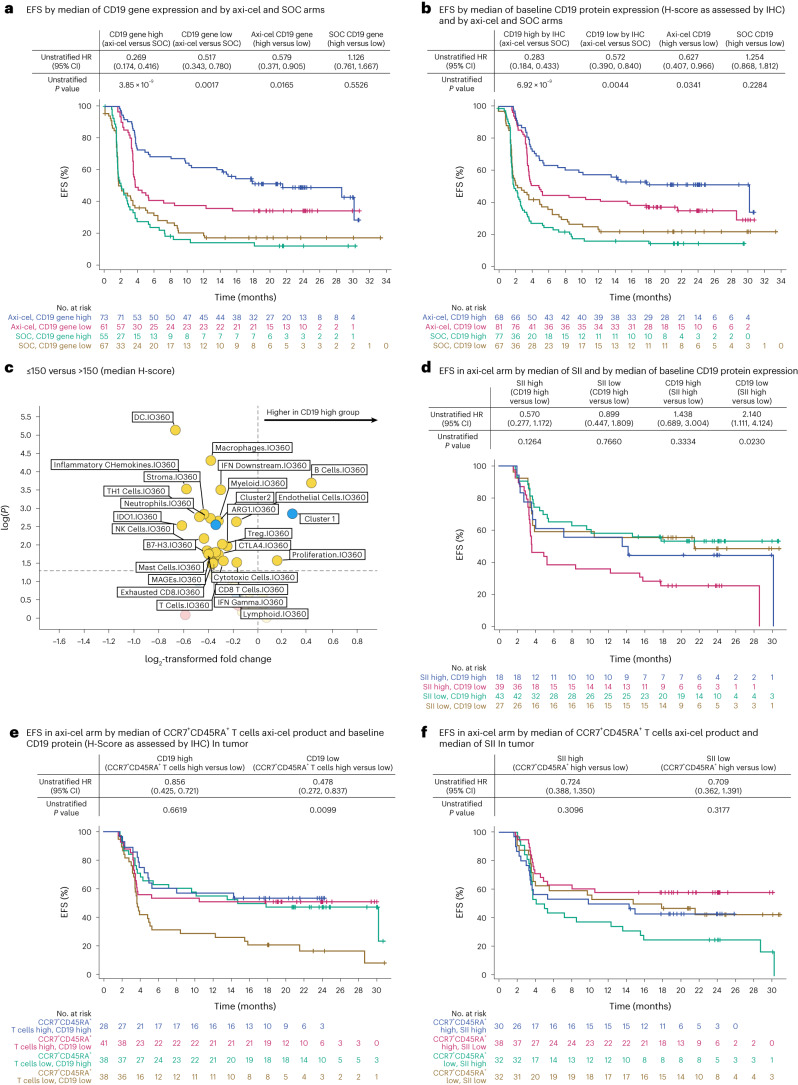
Patients treated with axi-cel who demonstrated improved EFS harbor higher CD19 gene expression and protein in the tumor. For all panels, patients who did not meet the criteria for an event had their data censored (tick marks). **a**, Kaplan–Meier estimate of EFS by CD19 gene expression and treatment arm (axi-cel versus SOC). **b**, Kaplan–Meier estimate of EFS by CD19 protein expression (H-score as assessed by IHC) and treatment arm (axi-cel versus SOC). **c**, Association between CD19 H-score and GES as a volcano plot. The plot presents descriptive *P* value and fold change of GES in patients with a median CD19 H-score ≤150 versus >150. Clusters 1 and 2 are shown in blue. The fold change is presented as log_2_((group one(/(group two)). Statistical analyses were conducted using Kruskal–Wallis test (numerical versus categorical). **d**, Kaplan–Meier estimate of EFS in the axi-cel group by SII and CD19 protein expression. **e**, Kaplan–Meier estimate of EFS in the axi-cel group by median of CCR7^+^CD45RA^+^ T cells in axi-cel product and CD19 protein (H-score) in tumor. **f**, Kaplan–Meier estimate of EFS in the axi-cel group by median of CCR7^+^CD45RA^+^ T cells in axi-cel product and SII in tumor. For panels **a**, **b** and **d**–**f**, unstratified Cox proportional hazards *P* values (two-sided) are presented.

CD19 protein expression (H-score) on malignant tumor B cells was correlated with CD19 gene expression and the B cell GES (Supplementary Fig. 3). Consistent with a role for the B cell GES in axi-cel-mediated efficacy (Fig. 1), CD19 gene and protein expression also correlated with axi-cel EFS (Fig. 4a,b). Axi-cel EFS was improved in patients with high (>median) CD19 gene and protein expression relative to those with lower expression (≤median; Fig. 4a,b). Axi-cel remained superior to SOC across CD19 gene expression subgroups (Fig. 4a,b). The objective response rate (ORR) in patients deemed CD19 negative by IHC (H-score <5) was 84.6% versus 66.7% in the axi-cel versus SOC arm, respectively (Supplementary Table 4, descriptive *P* = 0.6299).

**Extended Data Fig. 5:**
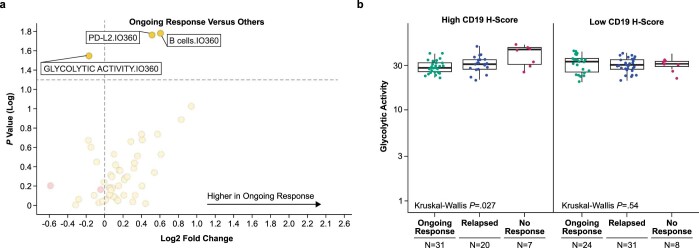
Failure to achieve durable response in patients with high CD19 H-score may be driven by a high glycolytic activity. Panel **a** shows association between ongoing response and IO360™ signatures as a volcano plot. Descriptive *P* value is reported over GES signature fold change in patients with ongoing response versus others. The fold change is shown as Log2([group one]/[group two]). Statistical analyses were conducted using Kruskal–Wallis test (numerical vs categorical). Panel **b** shows glycolytic activity (IO360) by response in the axi-cel arm for patients with high CD19 H-score (>median; left; n = 31, ongoing response; n = 20, relapsed; n = 7, no response) and low CD19 H-score ( ≤ median; right; n = 24, ongoing response; n = 31, relapsed; n = 8, no response). Box plots show Q1, median, and Q3, and the lower and upper whiskers show Q1-1.5(IR) and Q3 + 1.5(IR), respectively; n values reflect the number of independent patients in each respective group. Two-sided *P* values were calculated per Kruskal-Wallis test and are reported. Axi-cel, axicabtagene ciloleucel; GES, gene expression signature; IR, interquartile range; Q, quartile.

Patients with lower CD19 protein expression (H-score ≤ median) harbored a more complex, immune-infiltrated TME enriched with several immunosuppressive features, including GES for regulatory T cells, T cell exhaustion, ARG1, indoleamine 2,3-dioxygenase 1 (IDO1), B7-H3, CTLA4, and macrophage and myeloid cells (Fig. 4c). The poorest EFS after axi-cel treatment was observed in patients with tumors that harbored both low CD19 protein expression and high SII, suggesting that both TME immunosuppression and target expression play a role in resistance to CAR T cell therapy (Fig. 4d). Conversely, in patients with higher CD19 protein expression (>median), lack of durable response was associated with increased glycolytic activity (Extended Data Fig. 5). CD19 expression did not associate with grade ≥3 cytokine release syndrome or neurological events (Supplementary Tables 2 and 3).

### A stem-like axi-cel product may overcome an unfavorable TME

Similar to evidence obtained in ZUMA-1 (third line)^6^, T cell immunophenotyping of ZUMA-7 axi-cel products suggested that less differentiated cells in the CAR T cell product (CCR7^+^CD45RA^+^ T cells) were associated with improved efficacy and survival^19,20^. Here, we further investigated whether an axi-cel product enriched in CCR7^+^CD45RA^+^ T cells, considered a naive-like T cell or stem memory phenotype^21^, may overcome the adverse effects of an unfavorable TME. Indeed, patients with relatively lower CD19 protein expression (H-score as assessed by IHC) showed improved EFS when there was a higher frequency of CCR7^+^CD45RA^+^ T cells in the product (Fig. 4e). Patients with relatively higher SII also showed a trend toward improved EFS with higher frequency of CCR7^+^CD45RA^+^ T cells in the product (Fig. 4f; descriptive *P* = 0.3096). Notably, the frequency of product CCR7^+^CD45RA^+^ T cells (Supplementary Fig. 4 for gating strategy) did not associate with tumor CD19 protein or gene expression, B cell GES or SII (Supplementary Table 5).

### High SPD and elevated LDH impact SOC outcomes

**Fig. 5:**
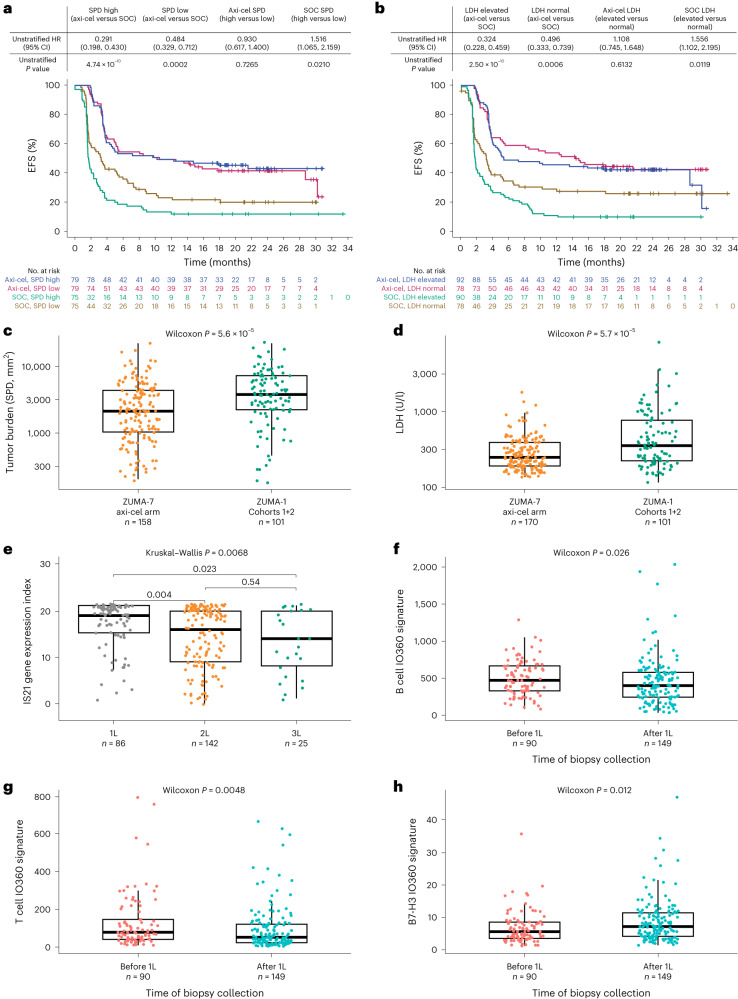
Axi-cel EFS was superior to SOC irrespective of SPD or LDH. **a**, Kaplan–Meier estimate of EFS by SPD and treatment arm (axi-cel versus SOC). **b**, Kaplan–Meier estimate of EFS by LDH and treatment arm (axi-cel versus SOC). For **a** and **b**, patients who did not meet the criteria for an event had their data censored (tick marks); unstratified Cox proportional hazards *P* values (two-sided) are presented. **c**,**d**, Tumor burden (SPD; **c**) and LDH (**d**) of ZUMA-7 (axi-cel arm; *n* = 158, tumor burden; *n* = 170, LDH) and ZUMA-1 phase 2 cohorts 1 + 2 patients (*n* = 101 for tumor burden and LDH). **e**, IS21 gene expression indices by line of therapy (*n* = 86, 1 L; *n* = 142, 2 L; *n* = 25, 3 L. **f**, B cell IO360 GES at initial diagnosis (*n* = 90) and after first-line therapy (*n* = 149). **g**, T cell IO360 GES at initial diagnosis (*n* = 90) and after first-line therapy (*n* = 149). **h**, B7-H3 IO360 GES at initial diagnosis (*n* = 90) and after first-line therapy (*n* = 149). For panels **c**–**h**, box plots show Q1, median and Q3, and the lower and upper whiskers show Q1 – 1.5 × IQR and Q3 + 1.5 × IQR, respectively; *n* values reflect the number of independent patients in each respective group. Two-sided *P* values were calculated per Wilcoxon test and are reported. 1L, first-line; 2L, second-line; 3L, third-line; IS, ImmunoSign.

Tumor burden, per sum of product diameters (SPD) and LDH levels—both known prognostic biomarkers in LBCL^3,6^—were evaluated. SPD was correlated with LDH (Spearman *R*, 0.42; *P* = 1.93 × 10^–14^). In the axi-cel arm, there was no significant association between outcome and SPD or LDH, whereas SOC outcomes were impacted by high SPD (>median) and elevated LDH (Fig. 5a,b). Axi-cel EFS was improved versus SOC for both high (HR = 0.29; *P* = 4.74 × 10^–10^) and low SPD (HR = 0.49; *P* = 0.0002) and when comparing elevated and normal LDH levels (HR = 0.32 and 0.50, respectively; *P* = 2.5 × 10^–10^ and *P* = 0.0006). EFS in axi-cel patients was not significantly associated with SPD (HR = 0.92; 95% CI, 0.60–1.34; *P* = 0.68) or LDH (HR = 1.1; 95% CI, 0.75–1.65; *P* = 0.61), but was worse in SOC patients with higher SPD (HR = 1.51; 95% CI, 1.06–2.15; *P* = 0.02) or higher LDH (HR = 1.56; 95% CI, 1.10–2.20; *P* = 0.01).

Consistent results with SPD were observed by logistic regression analyses around the probability of achieving complete response (Supplementary Fig. 5). A significant (*P* = 0.0029) association between SPD and complete response was observed only for the SOC arm (Supplementary Fig. 5). In third-line LBCL (ZUMA-1 pivotal cohorts 1 + 2), there was a strong correlation between SPD or LDH and ongoing response in axi-cel-treated patients^6^. Given that a similar correlation was not observed in second-line LBCL (ZUMA-7 axi-cel arm; Fig. 5a,b), it was hypothesized that differences in SPD, LDH, TME characteristics and other prognostic parameters might account for the differences between ZUMA-1 and ZUMA-7 by shifting the relative impact of these factors. Indeed, a comparison of SPD or LDH between ZUMA-1 (cohorts 1 + 2) and the ZUMA-7 axi-cel arm demonstrated that patients in ZUMA-7 had overall lower median SPD and LDH versus patients in ZUMA-1 (Fig. 5c,d). The range of SPD was similar, while the range of LDH was higher in ZUMA-7.

**Extended Data Fig. 6:**
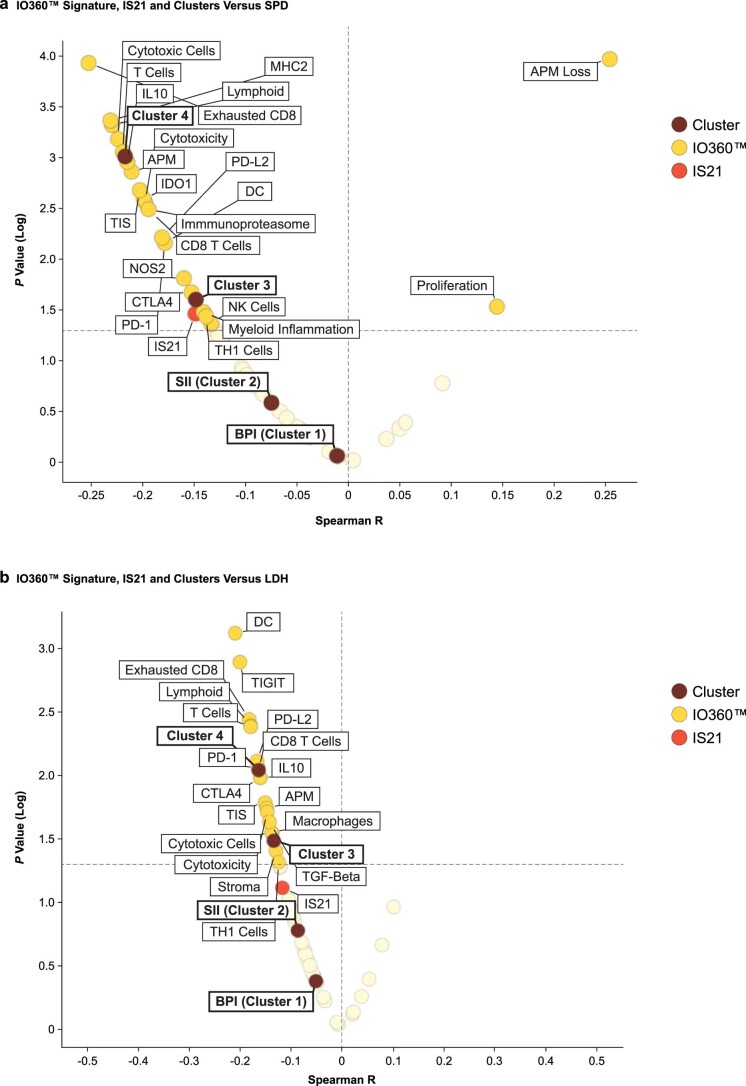
High SPD or LDH tumors were less immune infiltrated. Panels **a** and **b** show the relation of SPD (Panel a) or LDH (Panel b) with GES, as indicated. The volcano plots present descriptive two-sided P value over Spearman’s R. APM, antigen presentation machinery; DC, dendritic cell; LDH, lactate dehydrogenase; IDO1, indoleamine 2,3-dioxygenase 1; IFN, interferon; IS, ImmunoSign; JAKSTAT, Janus kinase signal transducer and activator of transcription; MHC, major histocompatibility complex; NK, natural killer; NOS, nitric oxide synthase; PD, programmed death; PD-L, programmed death-ligand; SPD, sum of product diameters; TGF, transforming growth factor; Th1, T helper type 1; TIS, tumor inflammation signature.

SPD and LDH negatively correlated with a number of NanoString IO360 signatures, and with clusters 3 and 4 (Extended Data Fig. 6), indicative of a less immune-infiltrated TME in high burden tumors. Indeed, high SPD positively correlated with APM loss, and negative correlations were found for MHC2, APM and several T cell and cytotoxic T cell subsets (Extended Data Fig. 6). These findings are consistent with ZUMA-1 (ref. ^10^), where high tumor burden was also associated with reduced immune infiltration.

To further assess whether axi-cel may overcome high tumor burden or high LDH in second line, the relationship between SPD and durable response in ZUMA-7 was explored on patients with SPD >median value from ZUMA-1 (3,721 mm^2^) or with LDH value twice the upper limit of normal (ULN) from ZUMA-7 (ULN; 390 U/l). Even with these increased thresholds, there was no association between SPD or LDH and responses in the axi-cel arm (Supplementary Fig. 6a,b). In addition, logistic regression analysis showed the lack of a strong association between SPD and responses to axi-cel among patients with high ZUMA-7 SPD (>median) or top-quartile of SPD (Supplementary Fig. 6c,d).

### Tumor immune contexture evolution through lines of therapy

Previously, GES of T cell functionality and trafficking into the TME, namely IS21, associated with complete response rate and PFS following third-line axi-cel treatment^10^. Here, IS21 correlated with lower tumor burden (Extended Data Fig. 6). Leveraging the timing of biopsy collection, we observed that IS21 expression was significantly lower in second-line and third-line therapy setting compared with first-line setting (Fig. 5e; *P* = 0.004 and *P* = 0.023 when comparing 1L versus 2L and 1L versus 3L). B cell and T cell IO360 GES also decreased (*P* = 0.026 and *P* = 0.0048, respectively) from initial diagnosis (before first line) to after first-line therapy (Fig. 5f,g), while immunosuppressive B7-H3 signature increased (Fig. 5h; *P* = 0.012).

**Extended Data Fig. 7:**
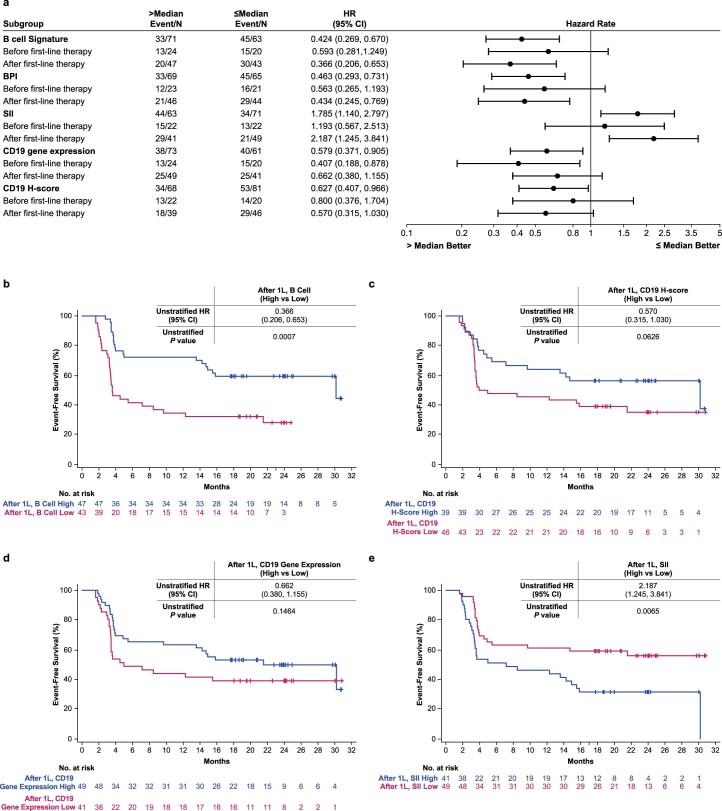
B-cell signature, BPI, CD19 expression and SII association with EFS following axi-cel treatment by timing of biopsy collection. Panel **a** shows the association of B-cell signature, BPI, CD19 expression as mRNA or H-score, or SII with EFS following axi-cel treatment, stratified by tumor biopsy collection timing: either before (initial diagnosis) or after 1 L treatment versus overall. For each subgroup, the number of independent patients with an event >median or ≤median are reported within the figure out of the total number of patients in each respective subgroup (ie, n with event/N total for the subgroup). Panel **b-e** show the Kaplan-Meier estimates of EFS for select biomarkers (as indicated), limited to patients with biopsy collected after 1 L treatment, before lymphodepletion chemotherapy (proximal to axi-cel treatment). Unstratified Cox proportional hazards *P* values (two-sided) are presented. For panels b-e, patients who did not meet the criteria for an event had their data censored (tick marks). axi-cel, axicabtagene ciloleucel; CI, confidence interval; EFS, event-free survival; HR, hazard ratio.

**Extended Data Fig. 8:**
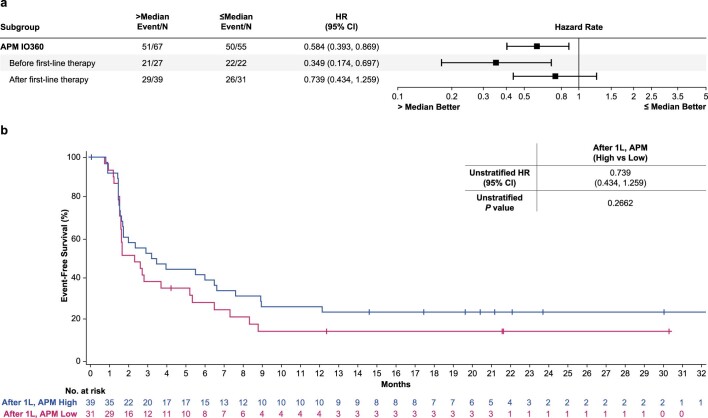
APM IO360™ signature association with EFS following SOC treatment by timing of biopsy collection. Panel **a** shows the association of APM IO360™ signature with EFS following SOC treatment stratified by tumor biopsy collection timing: either before (initial diagnosis) or after 1 L treatment versus overall. For each subgroup, the number of independent patients with an event >median or ≤median are reported within the figure out of the total number of patients in each respective subgroup (ie, n with event/N total for the subgroup). Panel **b** shows the Kaplan-Meier estimate of EFS by APM, limited to patients with biopsy collected after 1 L treatment. Unstratified Cox proportional hazards *P* value (two-sided) is presented. Patients who did not meet the criteria for an event had their data censored (tick marks). CI, confidence interval; EFS, event-free survival; HR, hazard ratio; SOC, standard of care.

**Extended Data Fig. 9:**
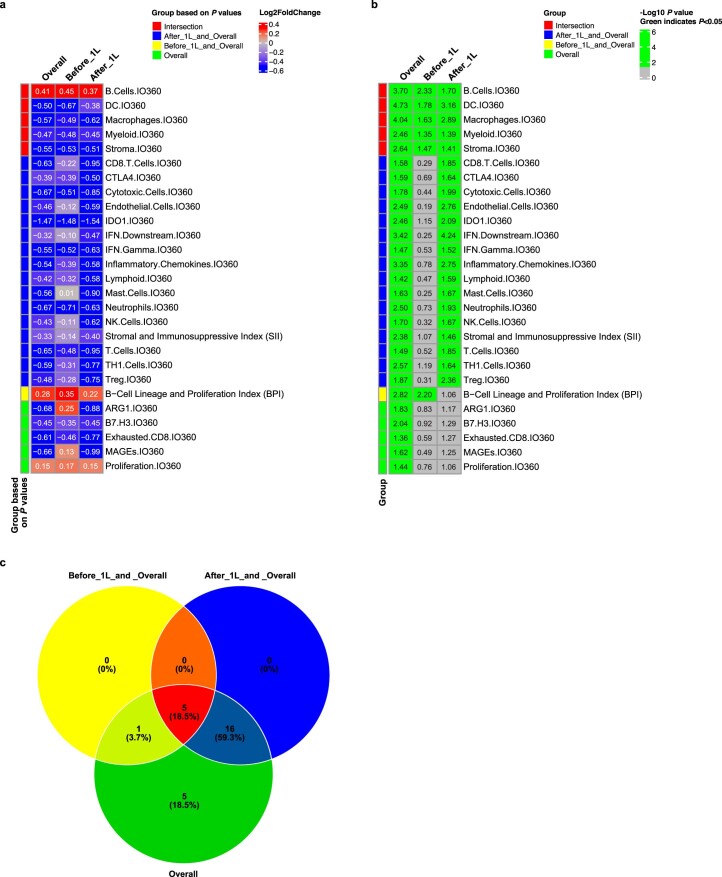
CD19 association with TME inflammation by timing of biopsy collection. Panels **a** and **b** show the fold change and descriptive two-sided *P* value (assessed via Wilcoxon test) for each GES that was significantly (*P* < 0.05) associated with CD19 H-score in at least one of the subgroups based on timing of biopsy collection, overall, before first-line therapy (initial diagnosis), or after first-line therapy. Panel **c** shows a venn diagram for the overlap of the significant associations across the 3 groups.

Subgrouping of the associative analyses described in the above sections, based on timing of biopsy collection (Supplementary Table 6), substantially reduces the number of patients for each group, limiting interpretability. Of note, more biopsies were collected after first line compared with before first-line therapy. Nevertheless, these analyses presented clear consistency for the associations of B cell signature, BPI, CD19 expression (mRNA or H-score) or SII with EFS following axi-cel treatment, regardless of the time of biopsy collection (Extended Data Fig. 7). With the sole exception of *CD19* mRNA, the predictive value of the GES seemed to increase when assessment was performed proximal to axi-cel treatment (after first line), which is particularly observed for the SII signature. Consistency was also observed in the SOC arm for the APM IO360 GES, albeit the predictive value of the GES seemed stronger when assessed at initial diagnosis (before first-line therapy; Extended Data Fig. 8). The negative correlation between CD19 H-score and stromal and inflammatory GES was observed regardless of the timing of biopsy collection (Extended Data Fig. 9). Regardless of timing of biopsy collection, B cell and proliferation GES increased consistently as CD19 H-score increased (H-score > median), while all other signatures, except ARG1 and melanoma antigen gene, decreased consistently as CD19 H-score increased.

### Molecular subgrouping did not impact outcome in axi-cel arm

**Fig. 6:**
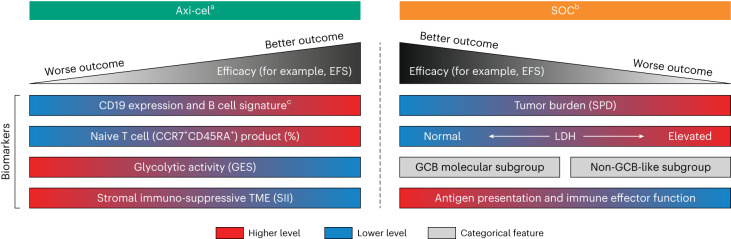
Summary of second-line biomarkers associated with efficacy. Biomarkers associated with efficacy in the axi-cel (left) and SOC (right) arms. Red represents higher levels and blue represents lower levels. Antigen presentation and immune effector function includes possible associations with macrophages, myeloid, APM, NK or CD8 T cells. ^a^Tumor burden (by SPD), LDH and GCB subgroup did not impact outcomes in the axi-cel arm. ^b^CD19 expression did not impact outcomes in the SOC arm. ^c^CD19 protein expression was measured by IHC, and B cell signature and *CD19* mRNA by NanoString.

**Extended Data Fig. 10:**
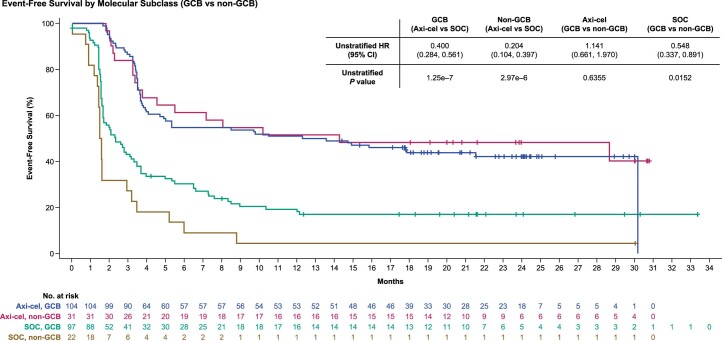
Axi-cel was not impacted by COO molecular subclass. Figure shows the Kaplan-Meier estimate of EFS by GCB status and treatment arm (axi-cel versus SOC). Patients who do not meet the criteria for an event had their data censored (tick marks). Unstratified Cox proportional hazards *P* values (two-sided) are presented. axi-cel, axicabtagene ciloleucel; CI, confidence interval; EFS, event-free survival; GCB, germinal center B cell-like; HR, hazard ratio; SOC, standard of care.

The prognostic value of molecular subgrouping into cell of origin (by gene expression analysis), germinal center B cell (GCB)-like, ABC-like and unclassified, were previously described in the context of first-line chemoimmunotherapy^22,23^. Considering the limited number of patients for ABC-like and unclassified subtypes in ZUMA-7 (ref. ^1^), these two categories were grouped together as non-GCB-like. Axi-cel EFS was similar for GCB-like and non-GCB-like subgroups and was superior to SOC (Extended Data Fig. 10; axi-cel, *P* = 0.6355 for GCB-like versus non-GCB-like; *P* = 1.25 × 10^–7^ and *P* = 2.97 × 10^–6^ for axi-cel versus SOC for GCB-like and non-GCB-like, respectively; Fig. 6 for representative model). Conversely, non-GCB status was associated negatively with SOC EFS (*P* = 0.0114). All four gene expression clusters (Fig. 3) did not associate with cell of origin by molecular subgroup (Extended Data Fig. 4). The latter is also represented by a principal component analysis (PCA) utilizing all genomic features significantly associated with clinical outcomes (ongoing response, EFS or DOR) for either arm of the study (Supplementary Fig. 7). Cluster 1 (BPI) and B cell-related genes contributed inversely to the principal components as compared with features associated with clusters 2, 3 and 4 that had more similar contributions to the components, supporting the identified clustering in Fig. 3a. While the clusters were differentially involved in distinct sections of the PCA plot, GCB status showed a diffuse pattern, supporting the independence of GCB status from the above clusters.

## Discussion

ZUMA-7 is the largest available clinical dataset in the CAR T cell therapy setting for second-line LBCL. Here, we explored the ZUMA-7 dataset to uncover tumor biomarkers associated with outcome (EFS, DOR, ongoing response, complete response, objective response) to CAR T cell therapy (axi-cel) or SOC (salvage chemotherapy/HDT-ASCT). We determined that outcomes to axi-cel or SOC are influenced differentially by malignant cell characteristics and the composition of the TME in the second-line setting, providing insights into the putative mechanisms driving responsiveness to these therapies. For instance, tumor GES representative of immune contextures, including the B cell signature and SII, a cluster enriched with stromal and immune-suppressive features, were associated positively and negatively, respectively, with CAR T cell therapy outcome. Notably, bulk tumor CD19 gene expression by NanoString IO360 and malignant cell CD19 protein expression (H-score as assessed by IHC) were correlated and associated positively with cell therapy outcome.

The analyses reported herein identified principal clusters of GES. The SII, which associated negatively with clinical outcome, could be reflective of an immune-suppressive TME, including myeloid-associated immune-suppressive and TGFβ-activated stromal genes. Within this immune contexture, CAR T cells may not sufficiently traffic to malignant cells or sustain a functional state^24^. Previous work demonstrated an association between myeloid cell infiltration and checkpoint ligand upregulation in LBCL, and additional investigation is warranted to determine whether the SII-classified tumors promote T cell dysfunction. In contrast, BPI was associated positively with HGBL/double-/triple-hit disease and a favorable clinical outcome. The latter high-risk subgroup was more likely to have high BPI indices, indicating a more uniform malignant B cell population with less diverse immune infiltration. Therefore, favorable clinical outcomes with axi-cel may be dependent on tumors that, albeit aggressive and highly proliferative, lack active cellular suppressive mechanisms, making them more sensitive to CAR T cell intervention.

A main finding of this study was the clear distinction between axi-cel and SOC biomarkers associated with outcome (Fig. 6). While the B cell GES and CD19 H-score associated positively with outcomes after axi-cel treatment, other TME immune features, including APM and dendritic cells associated positively with outcomes after SOC. This reinforces the mechanistic distinction between direct antigen engagement of CD19 by the CAR under axi-cel, versus co-opting the endogenous immunity against tumor epitopes (dependent on antigen processing and presentation mechanisms^25^) under SOC. Notably, outcomes with axi-cel were improved versus those with SOC for all presented biomarker subgroups.

The association between CD19 protein expression (H-score) on malignant cells and outcome corroborated recent results from Spiegel et al. using flow cytometry in a smaller dataset (*n* = 15)^26^. Further investigation is warranted; extrapolation of these findings using real-world clinical testing for CD19 is not substantiated given the carefully controlled nature of the real-time fresh tumor quantitative flow assay by Spiegel et al. and the centralized and standardized nature of our IHC assay.

Axi-cel demonstrated improved EFS over SOC regardless of CD19 expression (bulk gene expression or H-score). Nevertheless, in the ZUMA-7 axi-cel arm, patients with lower CD19 H-score presented a more complex, immune-infiltrated TME, underscoring that the relatively shorter EFS of axi-cel in patients with lower CD19 protein expression may be dependent not only on suboptimal target expression, but also on concurrent and confounding immune contexture features. In fact, low CD19 H-score associated with SII, which associated negatively with axi-cel EFS, and the association between CD19 H-score and EFS seemed confined mostly to patients having a high SII index.

The four clusters of TME signatures described herein and the associations of clusters 1 (BPI) and 2 (SII) with CAR T cell therapy outcomes have not been reported previously. Alizadeh et al. reported that the overall gene expression profile of a complex diffuse LBCL (DLBCL) lymph node biopsy can be approximated by a collection of related GES, revealing cell-of-origin GCB and ABC prognostic subgroups^27^. Similar conclusions were presented by Rosenwald et al.^28^, where an unclassified molecular subgroup was identified in addition to GCB and ABC groups. Further genomic subgrouping of GCB and ABC have also been established by Chapuy et al.^29^. Cell of origin may itself have predictive value, where more intensive chemoimmunotherapy can be considered for the worse prognosis ABC subtype^30^. In our analyses, cell of origin had a predictive value for SOC therapy, but lacked predictive value for CAR T cell therapy, with axi-cel showing similar efficacy for either GCB or non-GCB subtypes, reflecting profound mechanistic differences between treatment modalities.

The four clusters of GES reported herein present biological features that are different from the GCB, ABC and type 3 (unclassified) signatures defined per cell of origin. There was no association between any of the four clusters with GCB or non-GCB status. Rosenwald et al. also uncovered additional DLBCL GES, named ‘proliferating cells,’ ‘reactive stromal and immune cells in the lymph node,’ and ‘major histocompatibility complex class II complex’^28^. While different methodologies prevent direct comparison, it seems likely that there is a partial overlap of the biological features represented by the two independent studies. For instance, cluster 2 (SII) here may have similarities with the ‘reactive stromal and immune cells in the lymph node’^28^.

Similarly, the ‘mesenchymal’ signature recently presented by Kotlov et al.^31^ in the context of first-line LBCL is expected to partly overlap with cluster 2 (SII) presented here. Kotlov et al. also found that the HGBL subtype was enriched in a ‘depleted (DP)’ microenvironment, which presented features of B cell proliferation and relatively lower infiltration of immune cells. Here, we present a strong association between cluster 1 (B cell proliferation) and HGBL. One could envision that cluster 1 presented here overlaps with the DP microenvironment of Kotlov et al.^31^. Notably, the authors found that the DP microenvironment associated with the worst PFS following R-CHOP (first-line therapy), while the mesenchymal subtype associated with the best PFS (among the four gene expression-defined TME). Here, we report potentially opposite outcomes with CAR T cell therapy, where the B cell proliferation and stromal clusters associated with best and worst outcome, respectively. These observations might have important implications with CAR T cell therapy and other therapeutics moving to earlier lines of treatment, as there may be response-predictive value to these TME signatures. Consistently, Steen et al. recently proposed up to five malignant B cell states and 39 immune cell states in LBCL, which could assemble into up to nine ecotypes (TME subtypes based on interactions between tumor and immune cell states), and showed predictive value for some of these tumor immune contextures^32^. Although several available therapies present distinct mechanisms of action, the best treatment option in LBCL may rely on stratification by deeper molecular characterization, including TME features and cancer cell mutational profile^10,31,33–35^.

Another main finding of this study was the shift in influential biomarkers, most notably tumor immune contexture, across lines of therapy, supporting earlier intervention with CAR T cell therapy. This, together with the possible impact of lines of therapy on CAR T cell product fitness^6,19,36^, may well explain the different landscape of predictive markers across different lines of therapy. As shown here, tumors exposed to fewer therapies had greater IS21, a GES previously associated with improved immune infiltration and outcome to axi-cel^10^, as well as greater B cell signature. This may be due to TME evolution and/or bias in patient survival and selection through lines of therapy. Altogether, because of favorable tumor characteristics and T cell fitness, it is possible that axi-cel would present a further improved therapeutic profile in first-line therapy. This scenario is consistent with results recently published from the ZUMA-12 study, which reported a 78% complete response rate and 89% ORR in first-line patients with high-risk LBCL treated with axi-cel^36^.

The results presented herein also demonstrate that axi-cel was superior to SOC with an even wider margin in patients with high SPD or elevated LDH—tumor-aggressiveness features with known negative prognostic value. There was a lack of an association between SPD or LDH and responses in the ZUMA-7 axi-cel arm. This differs from previous observations in third-line LBCL (ZUMA-1)^6^. While lower median SPD and LDH in ZUMA-7 versus ZUMA-1 might account for these differences, ZUMA-7 enrolled many patients with substantial tumor burden and elevated LDH, and the range of SPD was similar between the two studies. Thus, in ZUMA-7, the lack of association between SPD and outcome may be due, at least partly, to favorable TME immune contextures in the second-line versus the third-line setting, as described above. On the other hand, SPD and LDH may not be the most informative prognostic metrics of tumor burden, and other approaches might prove more useful, including metabolic tumor volume, as previously reported in both second-line and third-line LBCL^37,38^. These observations suggest that a more favorable TME immune contexture in second-line LBCL may enable CAR T cell therapy to overcome large tumor burden.

While patients with reduced B cell signature and less favorable immune TME showed a poorer clinical outcome, a key question is whether actionable product characteristics may help overcome such unfavorable features. As presented herein, a CAR T cell product enriched in the CCR7^+^CD45RA^+^ T cell phenotype may improve outcomes in patients with lower CD19 protein expression and higher immunosuppressive features.

This study had certain limitations. As the analyses herein are exploratory, conclusions drawn from these data will require further confirmation in an independent validation cohort. The number of patients included in each analysis varied due to the availability of data from ZUMA-7 and ZUMA-1 cohorts 1 + 2. While no statistically significant associations between sex and efficacy were detected in axi-cel or SOC arms, the detection of minor, yet significant, interactions would require a much larger sample size than currently available. Future studies should also gather spatial information to better understand tumor immune cell contexture.

Knowledge of the immune contexture is essential for understanding mechanisms of action and likelihood of prolonged response to CAR T cell therapy^18,39^. In addition to SPD, metabolic tumor volume, LDH and target (CD19) expression, measurements of tumor immune contexture using Immunoscore, IS21 (ref. ^36^), B cell, as well as stromal and immunosuppressive gene signatures, are emerging as important and interrelated determinants of durable responses to axi-cel intervention. Collectively, these observations may help inform studies evaluating patient management based on tumor biology/biomarkers and design of next-generation therapeutics.

## Methods

### Inclusion and ethics

Studies were approved by the institutional review board at each study site and were conducted in accordance with the Good Clinical Practice guidelines of the International Conference on Harmonization^1,40^. Patients provided written informed consent for samples to be collected and analyzed. Financial compensation was not provided to patients.

### Patient samples and efficacy readouts

Evaluable samples from patients in the safety analysis sets of ZUMA-7 (NCT03391466; *n* = 170) and ZUMA-1 cohorts 1 + 2 (NCT02348216; *n* = 101) were analyzed (Supplementary Tables 7 and 8). Covariates were overall uniform among the analysis subgroups. Clinical data from ZUMA-7 were collected using Medidata Rave from 77 sites worldwide. Between 25 January 2018, and 4 October 2019, 359 patients underwent randomization. The number of patients included in each analysis varies based on data availability; for clarity, the specific *n* values are included in each figure. The safety analysis set of ZUMA-7 was defined as randomized patients who received at least one dose of axi-cel or SOC. The safety analysis set of ZUMA-1 was defined as all patients treated with any dose of axi-cel.

ZUMA-7 efficacy endpoints (ORR, best response, EFS, DOR and ongoing response) utilized the primary analysis data cutoff date^1^. EFS was defined as time from randomization to the earliest date of disease progression per Lugano Classification^41^, commencement of new lymphoma therapy or death from any cause. Ongoing response was defined as patients who were in ongoing response (complete response or partial response) by the ZUMA-7 primary analysis data cutoff date^1^. Progression after response was defined as patients who achieved a complete response or partial response and subsequently experienced disease progression. Patients who achieved stable disease or progressive disease as best response were included within the category of no response^6^. To contextualize select findings, data from patients with evaluable samples in ZUMA-1 pivotal cohorts 1 + 2 were included with a minimum follow-up of 60 months.

### Analysis of GES

Tumor biopsy collection and processing of formalin-fixed paraffin-embedded biopsy specimens was similar between ZUMA-1 (ref. ^10^) and ZUMA-7. Gene expression data were collected from tumor biopsies via a central laboratory. Wet laboratory analysis of NanoString IO360 was performed at Neogenomics. Raw data were transferred to NanoString for calculation of the IO360 scores and cell-of-origin status (GCB versus non-GCB). All correlative analyses were formed at Kite, a Gilead Company. Analyses were reproduced by at least two independent contractors and further analyzed for correctness. In ZUMA-7, biopsy collection was based on availability with collections of either archival biopsy from initial diagnosis or freshly collected before ZUMA-7 lymphodepleting chemotherapy (when archival was not available, not paired, Supplementary Table 3). Gene expression and molecular subgroup analysis were performed by leveraging the NanoString PanCancer IO360 Panel and Lymphoma Subtyping Test. Predefined GES from NanoString (proprietary algorithm; https://nanostring.com/products/ncounter-assays-panels/oncology/pancancer-io-360/) were analyzed for clustering and association with efficacy readouts. Unsupervised clustering of GESs was performed in TIBCO Spotfire (v.11.4.3) using the calculated hierarchical clustering method (unweighted pair group method with arithmetic mean; distance measure-Euclidean, ordering weight-average value, empty value replacement method-constant value, replace with 0 and normalization-none). IS21, a predefined GES of T cell infiltration and function, was calculated from gene expression values of the PanCancer IO360 panel using a proprietary algorithm from Veracyte^10^. For individual gene expression values, Nanostring RCC and RLF files were imported on nsolver Analysis software (v.4.0). Raw data were further analyzed with nCounter Advanced Analysis (v.2.0.134) and normalized linear counts output were used for all further analysis. Based on the PanCancer IO360 panel, expression of individual genes, cell subtypes within the TME and their association with clinical outcome were investigated.

### Analysis of CD19 expression level

CD19 protein expression level was measured by IHC using a validated assay at NeoGenomics^42^. Hematoxylin and eosin staining allowed formalin-fixed paraffin-embedded tissue evaluation for tumor content and block quality controls. Slides were scanned with an Aperio AT2 slide scanner to generate digital images at ×20 magnification. A trained pathologist identified the tumor area and provided qualitative and semiquantitative assessments. IHC staining was performed using tissue sections and an automated immunostainer (DAKO). IHC staining for CD19 (LE-CD19, cytoplasmic domain) was scored by composite H-score. H-scores were calculated as a product of IHC intensity (scale 1–3) multiplied by the percentage of tumor cells at a given intensity (0–100%) by central pathology review. IHC staining with H-score <5 was assigned as ‘negative’; 5–300 was assigned as ‘positive’ for the purpose of data quantification.

### Analysis of product attributes

Product T cell phenotypes and other product attributes were assessed at Kite, a Gilead Company, by flow cytometry based on CCR7 and CD45RA expression (NanoString PanCancer IO360 Panel). The gating strategy to derive the T cell phenotypes is summarized in Supplementary Fig. 4. Additional product characterization of costimulatory (CD27, CD28) and activation and exhaustion markers (PD-1, TIM-3, LAG3) was performed by flow cytometry using a validated assay at CellCarta.

### Analysis of tumor burden

Tumor burden was estimated as the sum of product diameters of up to six target lesions per Cheson 2007 criteria^43^, assessed by central review. LDH was quantified at each site’s clinical laboratory, as previously described^1^. LDH was reported by each site as elevated (≥reference range) or nonelevated (<reference range) for the local laboratory.

### Association analysis and related statistics

Biomarkers from exploratory endpoints were analyzed for associations with each other and with efficacy endpoints. Spearman’s rank-order correlation was used to evaluate association between analytes. Kaplan–Meier plots and Cox regression were used to evaluate association between biomarkers and time-to-event endpoints. Wilcoxon rank sum test and logistic regression were used to evaluate the relationship between biomarkers and binary outcomes. Kruskal–Wallis tests were used to evaluate association between biomarkers and categorical endpoints. For these post hoc analyses, all *P* values were descriptive and *P* < 0.05 was considered significant. No adjustments for multiplicity testing were performed. Covariates were subdivided into subgroups by median value, quartile values, or as indicated (for example, SPD value of 3,721 mm^2^). Plots were generated using TIBCO Spotfire (v.11.4.3), SAS (v.8.3), R (v.4.2.3) or GraphPad Prism (v.8).

### PCA of outcome associated genomic features

PCA was performed utilizing all genomic features derived from NanoString expression profiling that were significantly associated with clinical outcomes in the axi-cel or SOC arm (*P* < 0.05; ongoing response, EFS or DOR). Genomic feature types included were IO360 signature scores, cluster scores derived from IO360 clusters, or genes included within any of the IO360 signatures. Subjects included in this analysis were those with NanoString expression profiling of pretreatment tumor biopsies which passed quality control *n* = 256). All PCA-related analyses and plots were performed in R v.4.2.2 (2022-10-31 ucrt). The FactoMineR (v.2.8) package was utilized to perform the PCA with the PCA function, default settings. The PCA loadings plot was generated using the fviz_pca_var() function from the factoextra package (v.1.0.7). All PCA patient dot plots were generated utilizing ggplot2 (v.3.4.2) functions and colors were represented with scale_color_manual for categorical feature overlays or scale_color_gradient2 for continuous variables where the feature values were log_10_-transformed and the midpoint of the color scaling was defined as the log_10_-transformed median value.

### Reporting summary

Further information on research design is available in the Nature Portfolio Reporting Summary linked to this article.

## Online content

Any methods, additional references, Nature Portfolio reporting summaries, source data, extended data, supplementary information, acknowledgements, peer review information; details of author contributions and competing interests; and statements of data and code availability are available at 10.1038/s41591-023-02754-1.

### Supplementary information

Supplementary Information Supplementary Tables 1–8 and Figs. 1–7.

Reporting Summary

## Data Availability

Kite is committed to sharing clinical trial data with external medical experts and scientific researchers in the interest of advancing public health. As such, Kite shares anonymized individual patient data (IPD) upon request or as required by law and/or regulation. Qualified external researchers may request IPD for studies of Kite or Gilead compounds approved in the USA and the European Union with a marketing authorization date on or after 1 January 2014 and are publicly listed on clinicaltrials.gov or the European Union-Clinical Trials Registry. For studies of newly approved compounds or indication, the IPD will be available for request 6 months after US Food and Drug Administration and European Medicines Agency approval. Such requests are at Kite’s discretion and are dependent on the nature of the request, the merit of the research proposed, availability of the data and the intended use of the data. If Kite agrees to the release of clinical data for research purposes, the requestor will be required to sign a data sharing agreement to ensure protection of patient confidentiality before the release of any data. Access can be requested by contacting medinfo@kitepharma.com and requests will be addressed within 60 days. The NanoString data from ZUMA-7 patients discussed in this publication will be deposited in the National Center of Biotechnology Information Gene Expression Omnibus (NCBI GEO) and will be accessible through the GEO Series with the following accession number and access code, respectively: GSE248835 and inmducmctjuxjsj.
