# Supplementary material for: Impact of tumor microenvironment on efficacy of anti-CD19 CAR T cell therapy or chemotherapy and transplant in large B cell lymphoma
Source: Nat Med. 2024 Jan 17;30(2):507–18. doi: 10.1038/s41591-023-02754-1 (PMC10878966; doi:10.1038/s41591-023-02754-1)
Supplement: Supplementary file 1 — Supplementary Tables 1–8 and Figs. 1–7. [file 41591_2023_2754_MOESM1_ESM.pdf]

nature medicine

Article

https://doi.org/10.1038/s41591-023-02754-1

Impact of tumor microenvironment on efficacy of anti-CD19 CAR T cell therapy or chemotherapy and transplant in large B cell lymphoma

In the format provided by the authors and unedited

SUPPLEMENTARY APPENDIX

Impact of Tumor Microenvironment on Efficacy of CD19 CAR T-Cell Therapy or Chemotherapy and Transplant in Large B-Cell Lymphoma

Locke* and Filosto*, et al.
*Contributed equally to this work

Table of Contents

Supplementary Table 1. NanoString IO360™ panel gene signatures. 2
Supplementary Table 2. Association of predictive biomarkers with grade ≥3 cytokine release syndrome following axi-cel treatment (safety analysis set). 4
Supplementary Table 3. Association of predictive biomarkers with grade ≥3 neurological events following axi-cel treatment (safety analysis set) 5
Supplementary Table 4. Comparison of objective response per central assessment by CD19 protein expression (H-score as assessed by IHC) positivity and arm (safety analysis set). 6
Supplementary Table 5. Association of CCR7+CD45RA+ T cells (as a percentage of viable CD3+ cells) with tumor CD19 gene expression, B-cell GES, or SII (safety analysis set). 7
Supplementary Table 6. Collection timing of pretreatment tumor biopsies by treatment arm. 8
Supplementary Table 7. Baseline characteristics of patients from ZUMA-7; based on data availability, as indicated. 9
Supplementary Table 8. Baseline characteristics of patients from ZUMA-1 Cohorts 1+2; based on data availability, as indicated. 12
Supplementary Figure 1. Nanostring IO360™ gene clustering reveals distinct TME immune contextures. 14
Supplementary Figure 2. EFS and DOR by median of cluster 3 and cluster 4 and treatment arm. 16
Supplementary Figure 3. CD19 H-score correlated with CD19 gene expression and B-cell IO360™ signature. 18
Supplementary Figure 4. Gating strategy used to derive T cell phenotypes. 19
Supplementary Figure 5. Logistic regression curves of responses by SPD. 20
Supplementary Figure 6. Increased thresholds for tumor burden (by SPD) and LDH did not impact axi-cel EFS. 21
Supplementary Figure 7. Principal Component Analysis of Gene Expression Derived Features Significantly Associated with Clinical Outcome 23

Supplementary Table 1. NanoString IO360™ panel gene signatures.

Cluster	Cell type signature, biological activity signature, or single gene signature	Gene name
1: B-Cell Lineage and Proliferation Index (BPI)	APM loss	***B2M***, ***TAP1***, *TAP2*, ***TAPBP***, ***HLA-A***, ***HLA-B***, ***HLA-C***
	B cells	*BLK, CD19, MS4A1, TNFRSF17, FCRL2, FAM30A, PNOC, SPIB, TCL1A*
	CD45	*PTPRC*
	Glycolytic activity	***AKT1***, ***HIF1A***, ***SLC2A1***, ***HK2***, ***TPI1***, ***ENO1***, ***LDHA***, ***PFKFB3***, *PFKM*, ***GOT1***, *GOT2*, ***GLUD1***, ***HK1***
	JAK-STAT loss	*JAK1*, ***JAK2***, ***IFNGR1***, ***IRF1***, ***IFNGR2***
	MSI predictor	*MLH1, MSH2, MSH6, PMS2, EPM2AIP1, RNLS, SFXN1,* ***SMAP1, SREBF1, TTC30A***, *TYMS, WDR76,* ***WNT11, EIF5AL1***
	Proliferation	*MKI67, CEP55, KIF2C, MELK, CENPF, EXO1, ANLN,* ***RRM2***, *UBE2C, CCNB1, CDC20*
2: Stromal and Immunosuppressive Index (SII)	Apoptosis	*AXIN1, BAD, BAX, BBC3, BCL2L1*
	Endothelial cells	*FAM124B, KDR, CLEC14A, CXorf36, ROBO4, MYCT1, CDH5, TIE1, BCL6B, PALMD, MMRN2*
	Hypoxia	*BNIP3L, MXI1, ADM, PLOD2, P4HA1, ALDOC, SLC2A1,* ***PDK1***, *P4HA2, BNIP3*
	MAGE	*MAGEA3/A6, MAGEA1, MAGEA12, MAGEA4, MAGEB2, MAGEC2, MAGEC1*
	Mast cells	*MS4A2, CPA3, HDC, TPSAB1/B2*
	MMR loss	***MLH1***, ***PMS2***, ***MSH2***, ***MSH6***
	Myeloid inflammatory	*CXCL1, CXCL3, CXCL2, CCL20, AREG, FOSL1, CSF3, PTGS2, IER3, IL6*
	Stroma	*FAP, COL6A3, ADAM12, OLFML2B, PDGFRB, LRRC32*
	Single gene signatures	*ARG1, CD276 (B7-H3), NOS2, TGFB1*
3	Dendritic cells	*CCL13, CD209, HSD11B1*
	IFN downstream	*IFI16, IFI27, IFI35, IFIH1, IFIT1, IFIT2, IFITM1, IFITM2, IRF1, APOL6, TMEM140, PARP9, TRIM21, GBP1, DTX3L, PSMB9, OAS1, OAS2, ISG15, MX1, IFI6, IFIT3, IRF9, STAT2*
	IFN-γ	*STAT1, CXCL9, CXCL10, CXCL11*
	Immunoproteasome	*PSMB8, PSMB9, PSMB10*
	Inflammatory chemokines	*CCL2, CCL3/L1, CCL4, CCL7, CCL8*
	Macrophages	*CD163, CD68, CD84, MS4A4A*

	MHC2	*HLA-DRB5, HLA-DPA1, HLA-DPB1, HLA-DQB1, HLA-DRA, HLA-DRB1, HLA-DMA, HLA-DOA*
	Myeloid	*ITGAM, TLR4, IL1B, CSF1R, CSF3R, TLR2, TLR1, ITGAX, HCK, TLR8, SLC11A1, CD47, CD14, CLEC4E, CLEC7A, FCAR, FCN1, LILRA5, LILRB2, LYZ, NFAM1, P2RY13, S100A8, S100A9, SERPINA1, SIRPA, SIRPB2, TREM1, CLEC5A, CSF1, CYBB, FCGR1A, MARCO, NLRP3, FPR1, FPR3, CCL3/L1, DAB2, OLR1, C5AR1,* ***TREM2****, MRC1, CEBPB*
	Neutrophils	*CSF3R, S100A12, CEACAM3, FCAR, FCGR3A/B, FPR1, SIGLEC5*
	NK cells	*NCR1, XCL1/2*
	NK CD56dim cells	*IL21R, KIR2DL3, KIR3DL1, KIR3DL2*
	Th1 cells	*TBX21*
	Treg	*FOXP3*
	Single gene signatures	*IL10, CD274 (PD-L1), TIGIT*
4	APM	*B2M, TAP1, TAP2, TAPBP, HLA-A, HLA-B, HLA-C*
	CD8 T cells	*CD8A, CD8B*
	Cytotoxic cells	*CTSW, GNLY, GZMA, GZMB, GZMH, KLRB1, KLRD1, KLRK1, PRF1, NKG7*
	Cytotoxicity	*GZMA, GZMB, GZMH, PRF1, GNLY*
	Exhausted CD8	*CD244, EOMES, LAG3, PTGER4*
	Lymphoid	*CXCL10, CXCR3, CX3CL1, PRF1, GZMK, GZMB, CD27, IL2RG, KLRK1, CTLA4, GZMH, CD3D, KLRB1, KLRD1, LCK, CD5, IRF4, CD8A, CD38, EOMES, GZMM, GNLY, IFITM1, IDO1,* ***MS4A1****, GZMA, CD2, CD3E, CD3G, CD40LG, CD6, CD7,* ***CD79A****, CD8B, CXCL11, CXCL13, CXCL9, HLA-DOB, IFNG, LAG3, LY9, PDCD1, TBX21, TIGIT, ZAP70, SLAMF7, CD96, PVR, STAT1, JAK1, JAK2, STAT2, IRF9, IGF2R, CD48, ICOS*
	T cells	*CD3D, CD3E, CD3G, CD6, SH2D1A, TRAT1*
	TIS	*CCL5, CD27, CD274, CD276, CD8A, CMKLR1, CXCL9, CXCR6, HLA-DQA1, HLA-DRB1, HLA-E, IDO1, LAG3, NKG7, PDCD1LG2, PSMB10, STAT1, TIGIT*
	Single gene signatures	*CTLA4, IDO1, PDCD1LG2 (PD-L2), PDCD1 (PD-1)*

Genes that are negatively associated with the cluster in which they are found are shown in **bold**. All other genes demonstrated a positive association with the cluster. APM, antigen processing machinery; IFN, interferon; JAK, Janus kinase; MAGE, melanoma antigen gene; MHC, major histocompatibility complex; MMR, mismatch repair; MSI, microsatellite instability; NK, natural killer; STAT, signal transducer and activator of transcription; Th1, T helper type 1; TIS, tumor inflammation signature; Treg, regulatory T cells.

Supplementary Table 2. Association of predictive biomarkers with grade ≥3 cytokine release syndrome following axi-cel treatment (safety analysis set).

	Axi-Cel (N=170)		
	Grade ≥3 (N=11)	Grade ≤2 or None (N=159)	*P* value
SII			
n	11	123	0.2335
Mean (SD)	23.53 (35.73)	18.02 (11.46)	
Median (Q1, Q3)	12.89 (10.89, 17.72)	14.97 (11.98, 19.54)	
Min, Max	8.22, 130.88	6.63, 89.68	
B-cell score			
n	11	123	0.3640
Mean (SD)	623.08 (520.68)	466.59 (288.17)	
Median (Q1, Q3)	566.13 (225.35, 635.61)	432.03 (252.83, 612.26)	
Min, Max	67.88, 2033.85	38.51, 1937.53	
CD19 H-score			
n	10	139	0.7469
Mean (SD)	152.00 (109.93)	143.35 (81.61)	
Median (Q1, Q3)	165.00 (70.00, 270.00)	140.00 (85.00, 200.00)	
Min, Max	0.00, 280.00	0.00, 300.00	
CD19 gene expression			
n	11	123	0.3902
Mean (SD)	2910.90 (3174.21)	1900.49 (1285.45)	
Median (Q1, Q3)	2177.57 (1045.75, 3863.80)	1685.21 (850.23, 2706.14)	
Min, Max	77.41, 11780.56	46.50, 6914.38	

Axi-cel, axicabtagene ciloleucel; max, maximum; min, minimum; Q1, quarter 1; Q3, quarter 3; SD, standard deviation; SII, Stromal and Immunosuppressive Index.

Supplementary Table 3. Association of predictive biomarkers with grade ≥3 neurological events following axi-cel treatment (safety analysis set).

	Axi-Cel (N=170)		
	Grade ≥3 (N=36)	Grade ≤2 or None (N=134)	*P* value
SII			
n	26	108	0.5158
Mean (SD)	24.24 (27.62)	17.08 (9.15)	
Median (Q1, Q3)	15.46 (12.19, 18.57)	14.45 (11.78, 19.00)	
Min, Max	8.22, 130.88	6.63, 54.30	
B-cell score			
n	26	108	0.6628
Mean (SD)	478.53 (382.52)	479.66 (296.88)	
Median (Q1, Q3)	494.58 (250.91, 574.44)	455.00 (268.37, 627.08)	
Min, Max	67.88, 2033.85	38.51, 1937.53	
CD19 H-score			
n	33	116	0.2210
Mean (SD)	127.58 (76.16)	148.59 (85.04)	
Median (Q1, Q3)	130.00 (70.00, 190.00)	142.50 (90.00, 220.00)	
Min, Max	0.00, 270.00	0.00, 300.00	
CD19 gene expression			
n	26	108	0.3518
Mean (SD)	1981.44 (2276.15)	1983.92 (1308.26)	
Median (Q1, Q3)	1550.91 (801.44, 2630.19)	1795.93 (996.47, 2801.72)	
Min, Max	77.41, 11780.56	46.50, 6914.38	

Axi-cel, axicabtagene ciloleucel; max, maximum; min, minimum; Q1, quarter 1; Q3, quarter 3; SD, standard deviation; SII, Stromal and Immunosuppressive Index.

Supplementary Table 4. Comparison of objective response per central assessment by CD19 protein expression (H-score as assessed by IHC) positivity and arm (safety analysis set).

	CD19 H-score positive (N=268)			CD19 H-score negative (N=25)		
	Axi-cel (N=136)	SOC (N=132)	*P* value	Axi-cel (N=13)	SOC (N=12)	*P* value
Response, n (%)	118 (86.8)	70 (53.0)	<.0001	11 (84.6)	8 (66.7)	0.6299
No response, n (%)	18 (13.2)	57 (43.2)		2 (15.4)	3 (25.0)	

Axi-cel, axicabtagene ciloleucel; SOC, standard of care.

Supplementary Table 5. Association of CCR7+CD45RA+ T cells (as a percentage of viable CD3+ cells) with tumor CD19 gene expression, B-cell GES, or SII (safety analysis set).

	Axi-Cel (N=170)		
	Number of evaluable patients	Spearman correlation estimate (95% CI)	*P* value
CD19 gene expression	132	-0.122 (-0.287, 0.050)	0.1610
B-cell score	132	-0.071 (-0.239, 0.101)	0.4193
SII	132	0.121 (-0.051, 0.286)	0.1658

Axi-cel, axicabtagene ciloleucel; GES, gene expression signature; SII, Stromal and Immunosuppressive Index.

Supplementary Table 6. Collection timing of pretreatment tumor biopsies by treatment arm.

	Axi-cel	SOC	Overall
Before 1L (Diagnosis)	44	49	93
After 1L	90	70	160
Unknown	0	3	3
Biopsy not provided	36	46	82
Total	170	168	338

1L, first-line therapy; Axi-cel, axicabtagene ciloleucel; SOC, standard of care.

Supplementary Table 7. Baseline characteristics of patients from ZUMA-7; based on data availability, as indicated.

	ZUMA-7					
	Safety analysis set (N=338)		CD19 H-score set N=293)		IO360™ set (N=256)	
	Axi-cel (N=170)	SOC (N=168)	Axi-cel (N=149)	SOC (N=144)	Axi-cel (N=134)	SOC (N=122)
Median age (range), y	58.5 (21, 80)	60.0 (29, 81)	59.0 (21, 77)	60.0 (29, 81)	57.5 (21, 77)	60.0 (29, 78)
≥65 years, n (%)	49 (29)	55 (33)	45 (30)	49 (34)	37 (28)	40 (33)
Male sex, n (%)	106 (62)	120 (71)	95 (64)	103 (72)	85 (63)	88 (72)
Race, n (%)						
American Indian or Alaska Native	0	1 (1)	0	1 (1)	0	1 (1)
Asian	11 (6)	8 (5)	11 (7)	8 (6)	8 (6)	5 (4)
Black or African American	9 (5)	6 (4)	7 (5)	6 (4)	8 (6)	5 (4)
Native Hawaiian or Other Pacific Islander	2 (1)	1 (1)	2 (1)	1 (1)	2 (1)	1 (1)
White	138 (81)	145 (86)	120 (81)	125 (87)	107 (80)	106 (87)
Other	10 (6)	7 (4)	9 (6)	3 (2)	9 (7)	4 (3)
Ethnicity, n (%)						
Hispanic or Latino	8 (5)	8 (5)	5 (3)	7 (5)	6 (4)	5 (4)
Not Hispanic or Latino	159 (94)	158 (94)	141 (95)	135 (94)	125 (93)	115 (94)
Not reported	3 (2)	2 (1)	3 (2)	2 (1)	3 (2)	2 (2)
ECOG score of 1, n (%)	78 (46)	74 (44)	69 (46)	67 (47)	64 (48)	60 (49)
Disease stage, n (%)						
I or II	40 (24)	33 (20)	36 (24)	29 (20)	30 (22)	21 (17)
III or IV	130 (76)	135 (80)	113 (76)	115 (80)	104 (78)	101 (83)
2L aaIPI of 2 or 3*, n (%)	73 (43)	75 (45)	62 (42)	63 (44)	60 (45)	55 (45)
Derived 2L aaIPI of 2 or 3, n (%)	78 (46)	75 (45)	67 (45)	63 (44)	65 (49)	55 (45)
Molecular subgroup per central laboratory, n (%)						
GCB-like	104 (61)	97 (58)	98 (66)	92 (64)	98 (73)	94 (77)
Activated B cell-like	14 (8)	9 (5)	13 (9)	8 (6)	14 (10)	9 (7)
Unclassified	17 (10)	13 (8)	16 (11)	11 (8)	17 (13)	12 (10)
Not applicable	10 (6)	16 (10)	8 (5)	14 (10)	5 (4)	6 (5)
Missing	25 (15)	33 (20)	14 (9)	19 (13)	0	1 (1)
Molecular subgroup per investigator, n (%)						
GCB-like	90 (53)	78 (46)	82 (55)	73 (51)	71 (53)	60 (49)
Non–GCB-like	44 (26)	49 (29)	39 (26)	39 (27)	36 (27)	33 (27)
Not tested	36 (21)	41 (24)	28 (19)	32 (22)	27 (20)	29 (24)

Response to 1L therapy at randomization, n (%)						
Primary refractory disease	123 (72)	123 (73)	107 (72)	107 (74)	91 (68)	86 (70)
Relapse at 12 mo after the initiation or completion of 1L	47 (28)	45 (27)	42 (28)	37 (26)	43 (32)	36 (30)
Derived response to 1L therapy at randomization, n (%)						
Primary refractory disease	124 (73)	125 (74)	108 (72)	109 (76)	92 (69)	89 (73)
Relapse at 12 mo after the initiation or completion of 1L	46 (27)	43 (26)	41 (28)	35 (24)	42 (31)	33 (27)
Disease type per central read or laboratory, n (%)						
DLBCL	121 (71)	119 (71)	118 (79)	118 (82)	103 (77)	94 (77)
High-grade B-cell lymphoma, not otherwise specified	0	1 (1)	0	1 (1)		1 (1)
High-grade B-cell lymphoma, including rearrangement of MYC with BCL2 or BCL6 or both	28 (16)	24 (14)	28 (19)	24 (17)	26 (19)	20 (16)
Not confirmed or missing data	16 (9)	19 (11)	0	0	2 (1)	4 (3)
Other	5 (3)	5 (3)	3 (2)	1 (1)	3 (2)	3 (2)
Disease type per investigator, n (%)						
TFL[†]	19 (11)	25 (15)	14 (9)	24 (17)	15 (11)	21 (17)
LBCL, not otherwise specified	103 (61)	110 (65)	93 (62)	92 (64)	84 (63)	79 (65)
T cell or histiocyte-rich LBCL	5 (3)	5 (3)	5 (3)	3 (2)	4 (3)	2 (2)
Epstein-Barr virus-positive DLBCL	2 (1)	0	2 (1)	0		
High-grade BCL, including rearrangement of MYC with BCL2 or BCL6 or both	40 (24)	25 (15)	34 (23)	24 (17)	30 (22)	18 (15)
Primary cutaneous diffuse large B-cell lymphoma, leg type	1 (1)	0	1 (1)	0	1 (1)	

Other	0	3 (2)	0	1 (1)		2 (2)
Prognostic market per central laboratory, n (%)						
High-grade BCL, double-/triple-hit	28 (16)	24 (14)	28 (19)	24 (17)	26 (19)	20 (16)
Double-expressor lymphoma	55 (32)	62 (37)	54 (36)	61 (42)	48 (36)	50 (41)
MYC rearrangement	13 (8)	7 (4)	13 (9)	7 (5)	11 (8)	7 (6)
Not applicable	71 (42)	68 (40)	54 (36)	52 (36)	49 (37)	45 (37)
Missing data	3 (2)	7 (4)	0	0		
CD19 positivity‡, n (%)						
Yes	136 (80)	132 (79)	136 (91)	132 (92)	119 (89)	107 (88)
No	13 (8)	12 (7)	13 (9)	12 (8)	8 (6)	8 (7)
Missing	21 (12)	24 (14)	0	0	7 (5)	7 (6)
Bone marrow involvement, n (%)						
Yes	16 (9)	14 (8)	15 (10)	12 (8)	12 (9)	12 (10)
No	154 (91)	154 (92)	134 (90)	132 (92)	122 (91)	110 (90)
Elevated LDH, n (%)	92 (54)	90 (54)	81 (54)	75 (52)	77 (57)	65 (53)
Median tumor burden (range), mm^2	2122.9 (181, 22538)	2135.1 (252, 20117)	2108.7 (181, 22538)	2167.3 (252, 20117)	2137.3 (181, 22538)	2326.2 (270, 20117)

*As determined by IxRS; †In ZUMA-7, values indicate large-cell transformation from FL; ‡CD19 positivity was assessed by immunohistochemical testing.

-, not available; 1L, first line; 2L, second line; 3L, third line; aaIPI, age-adjusted International Prognostics Index; ASCT, autologous stem cell transplantation; Axi-cel, axicabtagene ciloleucel; BCL, B cell lymphoma; DLBCL, diffuse large B cell lymphoma; ECOG, European Cooperative Oncology Group; FL, follicular lymphoma; GCB-like, germinal center B cell-like; IPI, International Prognostics Index; IxRS, interactive voice or Web-based response system; mo, months; LBCL, large B cell lymphoma; LDH, lactate dehydrogenase; Non–GCB-like; non-germinal center B cell-like; PMBCL, primary mediastinal large B cell lymphoma; SOC, standard of care; y, years; TLF, transformed follicular lymphoma.

Supplementary Table 8. Baseline characteristics of patients from ZUMA-1 Cohorts 1+2; based on data availability, as indicated.

	ZUMA-1 Cohorts 1+2	
	Cohort 1+2 (N=101)	Pre-3L biopsy (N=25)
Median age (range), y	58.0 (23, 76)	60.0 (27, 76)
≥65 years, n (%)	24 (24)	6 (24)
Male sex, n (%)	68 (67)	15 (60)
Race, n (%)		
American Indian or Alaska Native	0	0
Asian	3 (3)	0
Black or African American	4 (4)	0
Native Hawaiian or Other Pacific Islander	0	0
White	87 (86)	23 (92)
Other	7 (7)	2 (8)
Ethnicity, n (%)		
Hispanic or Latino	18 (18)	5 (20)
Not Hispanic or Latino	83 (82)	20 (80)
Not reported	0	0
ECOG score of 1, n (%)	59 (58)	13 (52)
Disease stage, n (%)		
I or II	15 (15)	8 (32)
III or IV	86 (85)	17 (68)
IPI, n (%)		
0	2 (2)	5 (20)
1	23 (23)	5 (20)
2	30 (30)	7 (28)
3	27 (27)	4 (16)
4	19 (19)	4 (16)
Molecular subgroup per central laboratory, n (%)		
GCB-like	49 (49)	12 (48)
Activated B cell-like	17 (17)	4 (16)
Unclassified	3 (3)	0
Not applicable	1 (1)	0
Missing	31 (31)	9 (36)
Molecular subgroup per investigator, n (%)		
GCB-like	46 (46)	14 (56)
Non–GCB-like	0	0
Not tested	0	0
Activated B cell-like	10 (10)	1 (4)

Unknown	44 (44)	10 (40)
Missing	1 (1)	0
Refractory subgroup, n (%)		
Primary refractory	3 (3)	1 (4)
Refractory to 2nd or greater line therapy	77 (76)	16 (64)
Relapse post ASCT	21 (21)	7 (28)
Disease type per central read or laboratory, n (%)		
DLBCL	69 (68)	21 (84)
DLBCL and small BCL	1 (1)	1 (4)
DLBCL to TFL and small BCL	1 (1)	0
PMBCL	3 (3)	1 (4)
TFL	9 (9)	0
Not confirmed or missing data	1 (1)	0
Other		
Disease type per investigator, n (%)		
DLBCL	77 (76)	20 (80)
PMBCL	8 (8)	2 (8)
TFL	16 (16)	3 (12)
CD19 positivity‡, n (%)		
Yes	74 (73)	22 (88)
No	8 (8)	2 (8)
Bone marrow involvement, n (%)		
Yes	8 (8)	1 (4)
No	87 (86)	24 (96)
Median tumor burden (range), mm^2	58.0 (23, 76)	60.0 (27, 76)

‡CD19 positivity was assessed by immunohistochemical testing.

1L, first line; 2L, second line; 3L, third line; aaIPI, age-adjusted International Prognostics Index; ASCT, autologous stem cell transplantation; Axi-cel, axicabtagene ciloleucel; BCL, B cell lymphoma; DLBCL, diffuse large B cell lymphoma; ECOG, European Cooperative Oncology Group; FL, follicular lymphoma; GCB-like, germinal center B cell-like; IPI, International Prognostics Index; IxRS, interactive voice or Web-based response system; mo, months; LBCL, large B cell lymphoma; LDH, lactate dehydrogenase; Non–GCB-like; non-germinal center B cell-like; PMBCL, primary mediastinal large B cell lymphoma; SOC, standard of care; y, years; TLF, transformed follicular lymphoma.

Supplementary Figure 1. Nanostring IO360™ gene clustering reveals distinct TME immune contextures.

Heatmap representation of Spearman rank-order correlation values between NanoString IO360™ GESs and genes used in the creation of the NanoString IO360™ GES scores. The NanoString IO360™ GES ordering is matched with the unsupervised clustering order from the heatmap in Figure 3a while unsupervised clustering was performed for the rows of the heatmap (genes). Row-side annotation coloring corresponds to the NanoString IO360™ GES(s) associated with each gene (row); in cases where a gene is associated with more than one signature, it was assigned to the Multiple IO360 Signatures annotation. Row names were colored purple if the gene was significantly associated with ongoing response versus others (response followed by progressive disease and no response) by the Wilcoxon rank sum test in either treatment arm. APM, antigen processing and presentation machinery; axi-cel, axicabtagene ciloleucel; ARG1, arginase1; BPI, B Cell and Proliferation Index; DC, dendritic cell; EFS, event-free survival; HGBL, high-grade B-cell lymphoma; HR, hazard ratio; IDO1, indoleamine 2,3-dioxygenase 1; IFN, interferon; IL10, interleukin 10; JAKSTAT, Janus kinase-signal transducer and activator of transcription; MAGE, melanoma antigen gene; MHC, major histocompatibility complex; MMR, mismatch repair; MSI, microsatellite instability; NK, natural killer; NOS, nitric oxide synthase; PD, programmed death; PD-L, programmed death-ligand; SII, Stromal and Immunosuppressive Index; SOC, standard of care; TGF, transforming growth factor; Th1, T helper type 1; TIS, tumor inflammation signature; Treg, regulatory T cell.

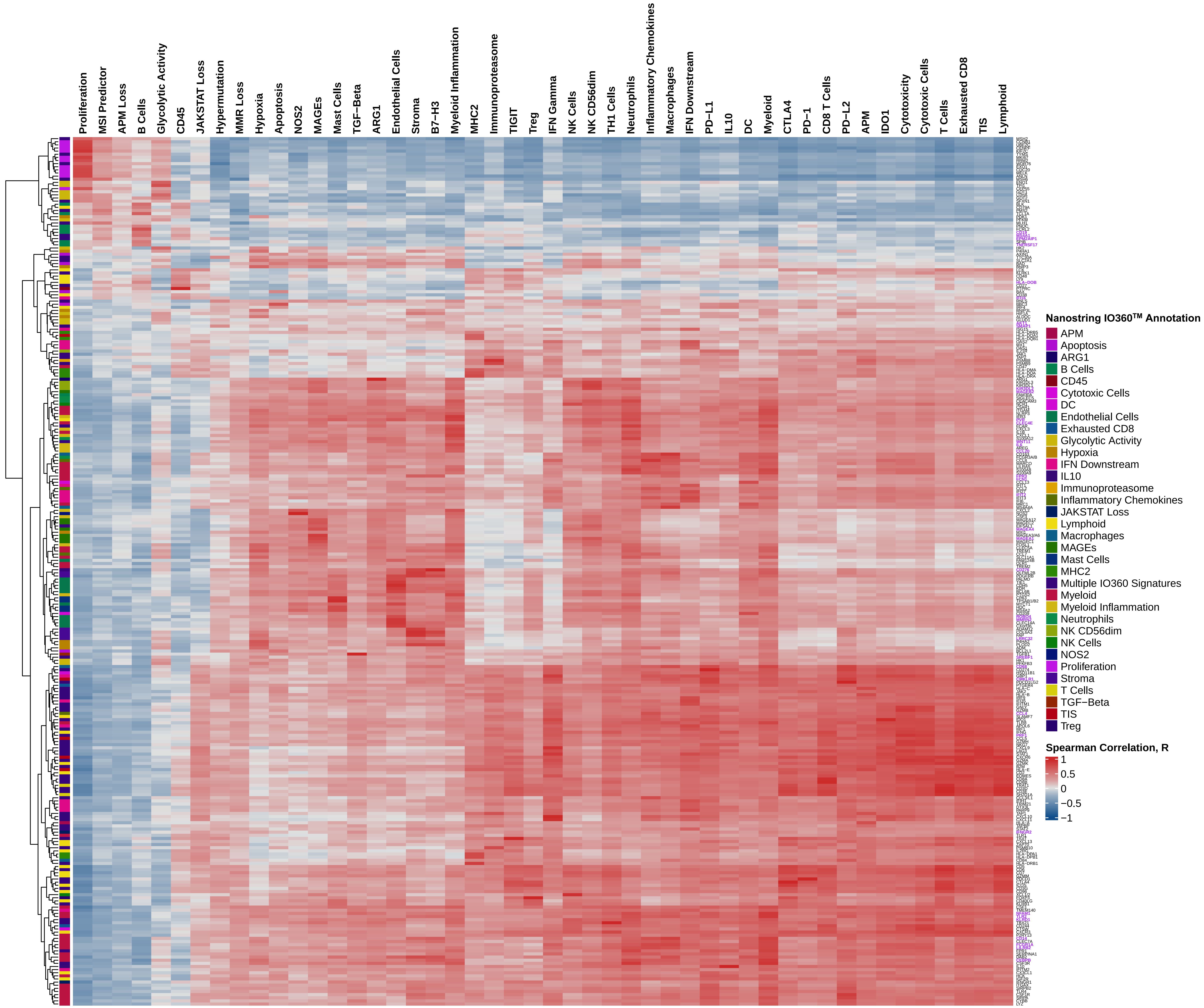
Proliferation
MSI Predictor
APM Loss
B Cells
Glycolytic Activity
CD45
JAKSTAT Loss
Hypermutation
MMR Loss
Hypoxia
Apoptosis
NOS2
MAGEs
Mast Cells
TGF−Beta
ARG1
Endothelial Cells
Stroma
B7−H3
Myeloid Inflammation
MHC2
Immunoproteasome
TIGIT
Treg
IFN Gamma
NK Cells
NK CD56dim
TH1 Cells
Neutrophils
Inflammatory Chemokines
Macrophages
IFN Downstream
PD−L1
IL10
DC
Myeloid
CTLA4
PD−1
CD8 T Cells
PD−L2
APM
IDO1
Cytotoxicity
Cytotoxic Cells
T Cells
Exhausted CD8
TIS
Lymphoid
Nanostring IO360TM Annotation
APM
Apoptosis
ARG1
B Cells
CD45
Cytotoxic Cells
DC
Endothelial Cells
Exhausted CD8
Glycolytic Activity
Hypoxia
IFN Downstream
IL10
Immunoproteasome
Inflammatory Chemokines
JAKSTAT Loss
Lymphoid
Macrophages
MAGEs
Mast Cells
MHC2
Multiple IO360 Signatures
Myeloid
Myeloid Inflammation
Neutrophils
NK CD56dim
NK Cells
NOS2
Proliferation
Stroma
T Cells
TGF−Beta
TIS
Treg
Spearman Correlation, R
1
0.5
0
−0.5
−1

Supplementary Figure 2. EFS and DOR by median of cluster 3 and cluster 4 and treatment arm.

Panels a and b show the Kaplan-Meier estimate of EFS by median of cluster 3 and cluster 4, respectively, and treatment arm (axi-cel versus SOC). Panels c and d show the Kaplan-Meier estimate of DOR by median of cluster 3 and cluster 4, respectively, and treatment arm (axi-cel versus SOC). For panels a-d, patients who did not meet the criteria for an event had their data censored (tick marks). Unstratified Cox proportional hazards *P* values (two-sided) are presented. Axi-cel, axicabtagene ciloleucel; DOR, duration of response; EFS, event-free survival; SOC, standard of care.

a EFS by Median of Cluster 3, High vs Low (Safety Analysis Set)

	Cluster 3 High (Axi-Cel vs SOC)	Cluster 3 Low (Axi-Cel vs SOC)	Axi-Cel Cluster 3 (High vs Low)	SOC Cluster 3 (High vs Low)
Unstratified HR (95% CI)	0.361 (0.235, 0.552)	0.385 (0.253, 0.586)	0.953 (0.610, 1.489)	1.044 (0.706, 1.543)
Unstratified *P* value	<.0001	<.0001	0.8336	0.8291

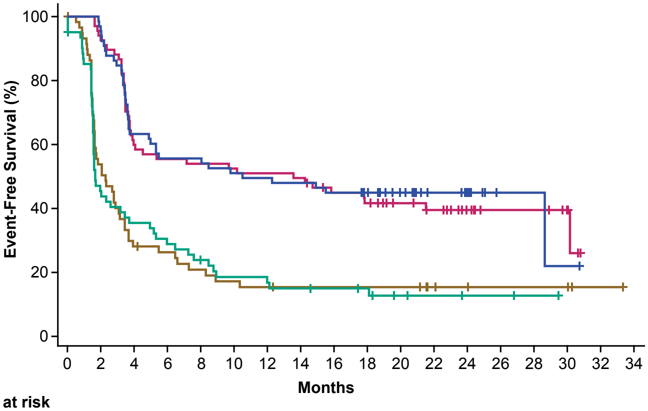

No. at risk	0	2	4	6	8	10	12	14	16	18	20	22	24	26	28	30	32	34
Axi-Cel, Cluster 3 High	66	64	42	37	37	34	33	32	30	27	21	15	11	2	2	1	0	
Axi-Cel, Cluster 3 Low	68	64	41	38	37	36	35	34	29	26	21	18	12	8	8	4	0	
SOC, Cluster 3 High	62	28	22	18	14	11	10	9	8	7	4	3	2	2	1	0		
SOC, Cluster 3 Low	60	32	17	15	12	10	9	8	8	8	8	5	4	3	3	3	1	0

b EFS by Median of Cluster 4, High vs Low (Safety Analysis Set)

	Cluster 4 High (Axi-Cel vs SOC)	Cluster 4 Low (Axi-Cel vs SOC)	Axi-Cel Cluster 4 (High vs Low)	SOC Cluster 4 (High vs Low)
Unstratified HR (95% CI)	0.369 (0.239, 0.569)	0.366 (0.241, 0.554)	0.873 (0.558, 1.368)	0.888 (0.601, 1.313)
Unstratified *P* value	<.0001	<.0001	0.5545	0.5511

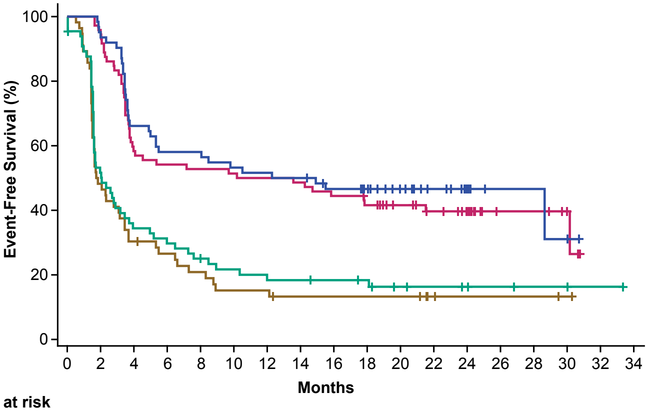

No. at risk	0	2	4	6	8	10	12	14	16	18	20	22	24	26	28	30	32	34
Axi-Cel, Cluster 4 High	62	59	41	36	36	33	32	31	27	24	18	13	8	3	3	2	0	
Axi-Cel, Cluster 4 Low	72	69	42	39	38	37	36	35	32	29	24	20	15	7	7	3	0	
SOC, Cluster 4 High	66	33	22	19	15	13	11	11	10	9	6	5	4	3	2	2	1	0
SOC, Cluster 4 Low	56	27	17	14	11	8	8	6	6	6	6	3	2	2	2	1	0	

c DOR by Median of Cluster 3, High vs Low (Safety Analysis Set)

	Cluster 3 High (Axi-Cel vs SOC)	Cluster 3 Low (Axi-Cel vs SOC)	Axi-Cel Cluster 3 (High vs Low)	SOC Cluster 3 (High vs Low)
Unstratified HR (95% CI)	0.827 (0.412, 1.659)	0.739 (0.401, 1.361)	0.854 (0.498, 1.465)	0.687 (0.324, 1.456)
Unstratified *P* value	0.5926	0.3309	0.5669	0.3269

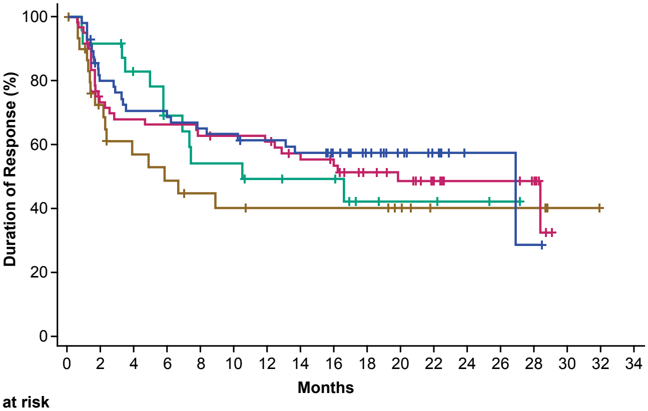

No. at risk	0	2	4	6	8	10	12	14	16	18	20	22	24	26	28	30	32	34
Axi-Cel, Cluster 3 High	56	43	38	37	35	34	32	30	26	21	15	9	2	2	1	0		
Axi-Cel, Cluster 3 Low	62	42	39	38	36	35	34	29	27	21	18	12	8	8	5	0		
SOC, Cluster 3 High	30	22	18	14	11	11	9	8	8	4	3	3	2	1	0			
SOC, Cluster 3 Low	35	19	14	12	10	9	8	8	8	8	6	3	3	3	3	1	0	

d DOR by Median of Cluster 4, High vs Low (Safety Analysis Set)

	Cluster 4 High (Axi-Cel vs SOC)	Cluster 4 Low (Axi-Cel vs SOC)	Axi-Cel Cluster 4 (High vs Low)	SOC Cluster 4 (High vs Low)
Unstratified HR (95% CI)	0.696 (0.363, 1.336)	0.813 (0.423, 1.561)	0.806 (0.469, 1.386)	0.878 (0.417, 1.849)
Unstratified *P* value	0.2758	0.5335	0.4358	0.7313

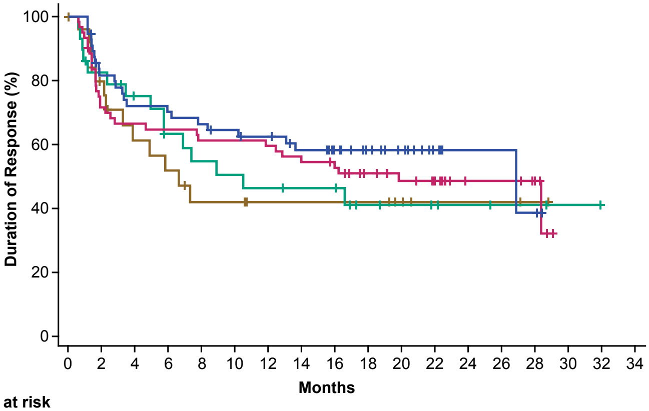

No. at risk	0	2	4	6	8	10	12	14	16	18	20	22	24	26	28	30	32	34
Axi-Cel, Cluster 4 High	56	43	38	37	35	33	31	27	23	18	13	7	3	3	2	0		
Axi-Cel, Cluster 4 Low	62	42	39	38	36	36	35	32	30	24	20	14	7	7	4	0		
SOC, Cluster 4 High	36	23	19	15	13	12	11	10	10	6	5	4	3	2	2	1	0	
SOC, Cluster 4 Low	29	18	13	11	8	8	6	6	6	6	4	2	2	2	1	0		

Supplementary Figure 3. CD19 H-score correlated with CD19 gene expression and B-cell IO360™ signature.

Panel a shows the relation between CD19 H-score and CD19 gene count, as assessed by Spearman rank-sum correlation. Panel b shows the association between CD19 H-score and B-cell signature (by IO360™), as assessed by Spearman rank-sum correlation. For both panels, statistical significance of the Spearman correlation coefficient (two-sided *P* value), as shown, was calculated. Blue dots represent individual patient data from 127 (panel A) and 127 (panel B) patients.

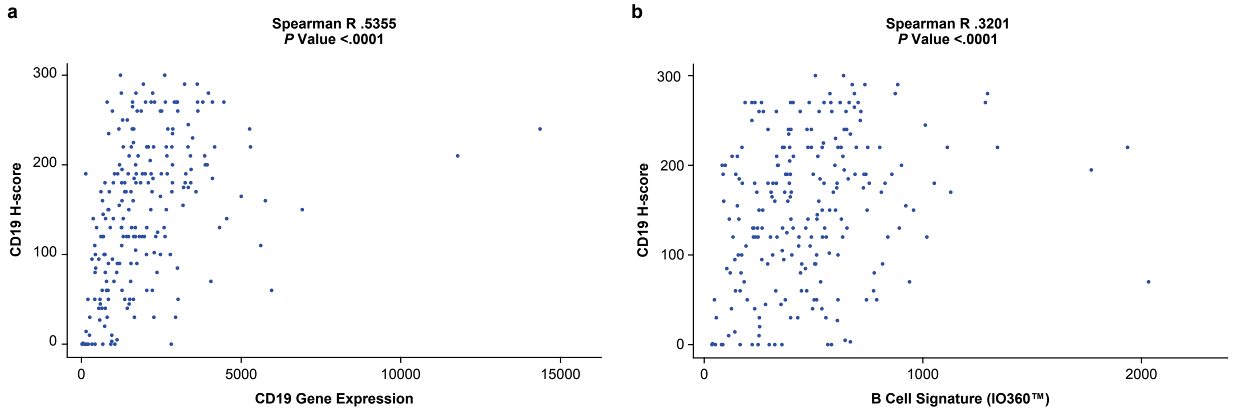

Supplementary Figure 4. Gating strategy used to derive T cell phenotypes.

Singlets/Viable/CD3+ cells were gated to identify T cells. Further subsets were identified by CD8, CCR7, CD45RA markers.

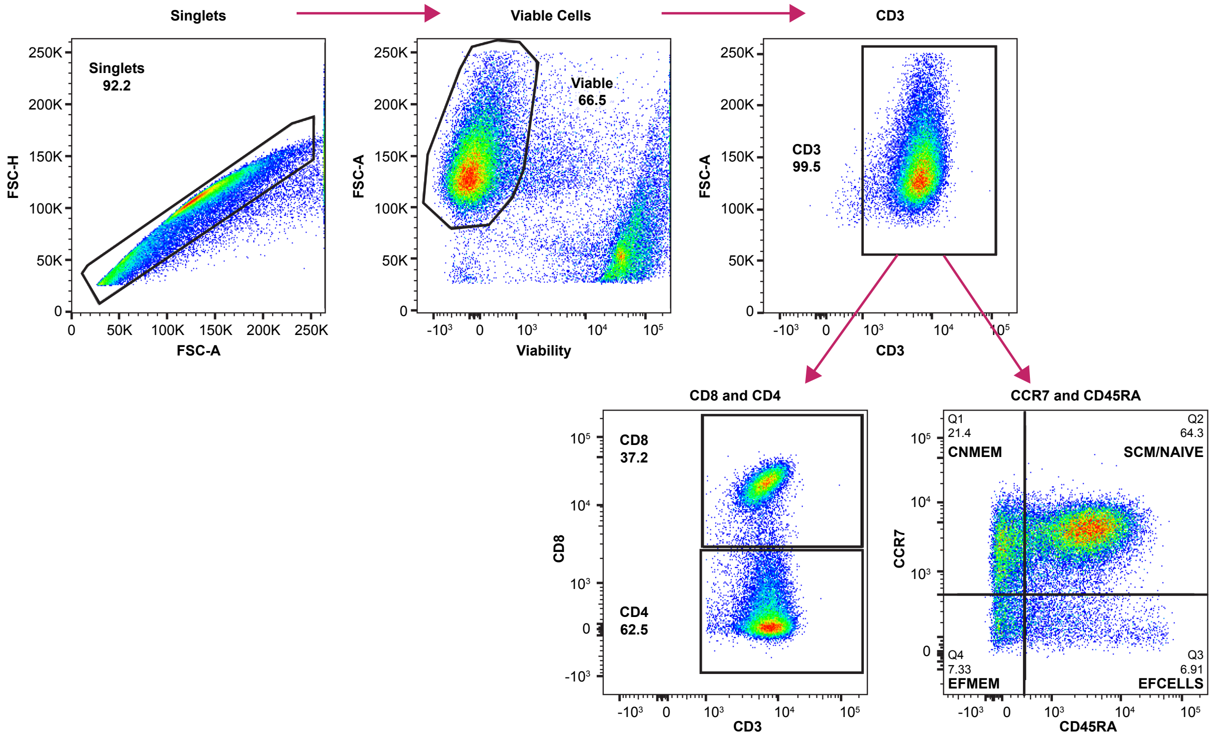

Supplementary Figure 5. Logistic regression curves of responses by SPD.

Panels a–f show logistic regression curves of responses in the axi-cel (Panels a–c) and SOC (Panels d–f) arms by tumor burden, as measured by SPD. Responses shown include best overall response per central assessment (Panels a and d), objective response per central assessment (Panels b and e), and ongoing response per central assessment (Panels c and f). For panels a-f, statistical significance was calculated via logistic regression and reported as two-sided *P* values; the blue ribbons represent the 95% confidence interval of the regression line. Axi-cel, axicabtagene ciloleucel; SOC, standard of care; SPD, sum of product diameters.

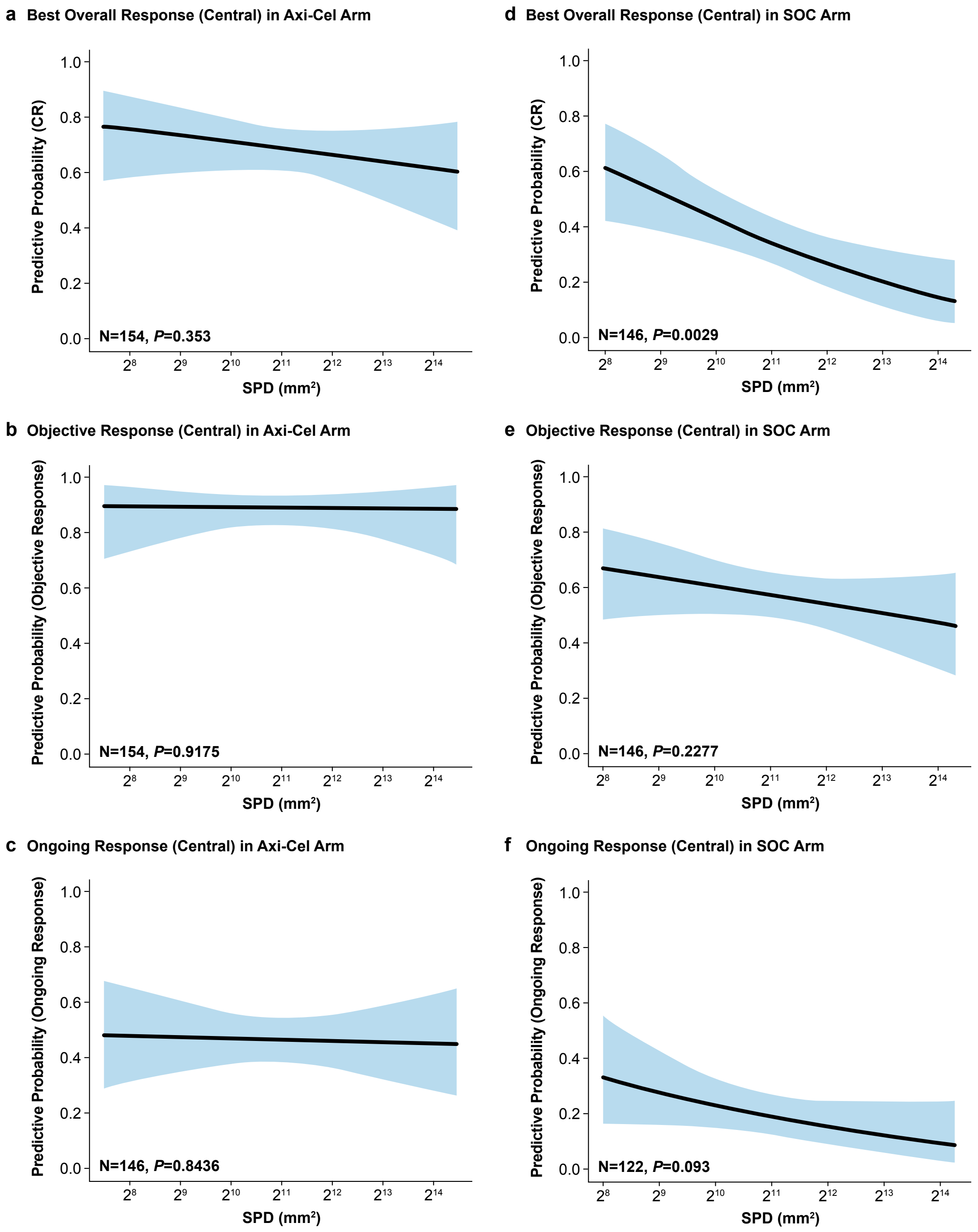

Supplementary Figure 6. Increased thresholds for tumor burden (by SPD) and LDH did not impact axi-cel EFS.

Panel a shows the Kaplan-Meier estimate of EFS by 3721 mm^2 as the cutoff value for high versus low SPD and treatment arm (axi-cel versus SOC). Panel b shows the Kaplan-Meier estimate of EFS by two times ULN (upper limit of normal) LDH and treatment arm (axi-cel versus SOC). For panels a and b, patients who did not meet the criteria for an event had their data censored (tick marks); unstratified Cox proportional hazards *P* values (two-sided) are presented. Panel c shows the logistic regression of ongoing response for ZUMA-7 axi-cel patients in the top quartile of SPD. Panel d shows the logistic regression of ongoing response for ZUMA-7 axi-cel patients with >median SPD. For panels c-d, statistical significance was calculated via logistic regression and reported as two-sided *P* values; the gray ribbons represent the 95% confidence interval of the regression line. axi-cel, axicabtagene ciloleucel; CI, confidence interval; EFS, event-free survival; HR, hazard ratio; LDH, lactate dehydrogenase; SOC, standard of care; SPD, sum of product diameters; ULN, upper level of normal.

a

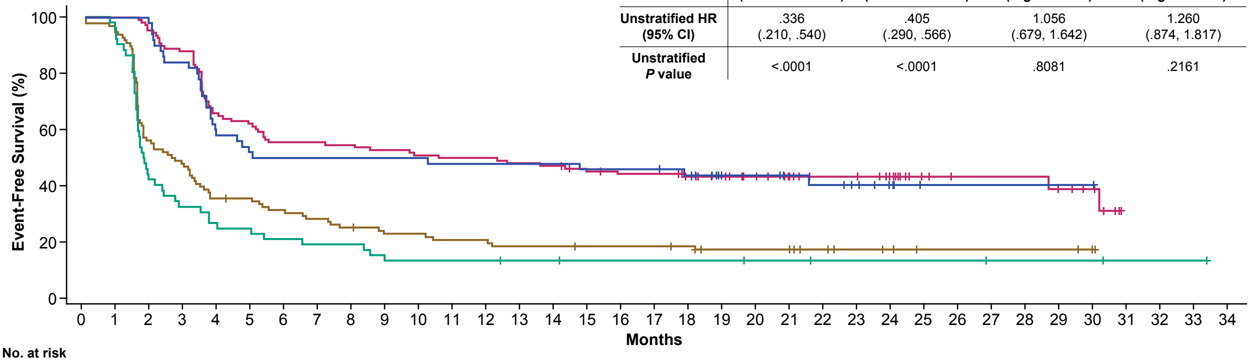

	SPD High (Axi-Cel vs SOC)	SPD Low (Axi-Cel vs SOC)	Axi-Cel SPD (High vs Low)	SOC SPD (High vs Low)
**Unstratified HR (95% CI)**	.336 (.210, .540)	.405 (.290, .566)	1.056 (.679, 1.642)	1.260 (.874, 1.817)
**Unstratified *P* value**	<.0001	<.0001	.8081	.2161

No. at risk

Months	0	1	2	3	4	5	6	7	8	9	10	11	12	13	14	15	16	17	18	19	20	21	22	23	24	25	26	27	28	29	30	31	32	33	34
Axi-Cel, SPD High	50	50	49	42	29	25	25	25	25	25	25	24	24	24	24	23	23	23	21	15	15	13	12	10	4	2	2	2	2	2	0				
Axi-Cel, SPD Low	108	108	103	95	70	66	60	60	59	57	55	54	54	52	51	47	45	45	41	37	32	28	25	24	21	12	10	10	10	8	6	0			
SOC, SPD High	52	47	22	17	13	12	11	10	10	7	7	7	7	6	6	5	5	5	5	5	4	4	3	3	3	3	3	2	2	2	2	1	1	1	0
SOC, SPD Low	98	92	54	46	35	33	29	27	23	21	21	19	18	17	17	16	16	16	15	12	12	11	8	6	5	3	3	3	3	3	1	0			

b

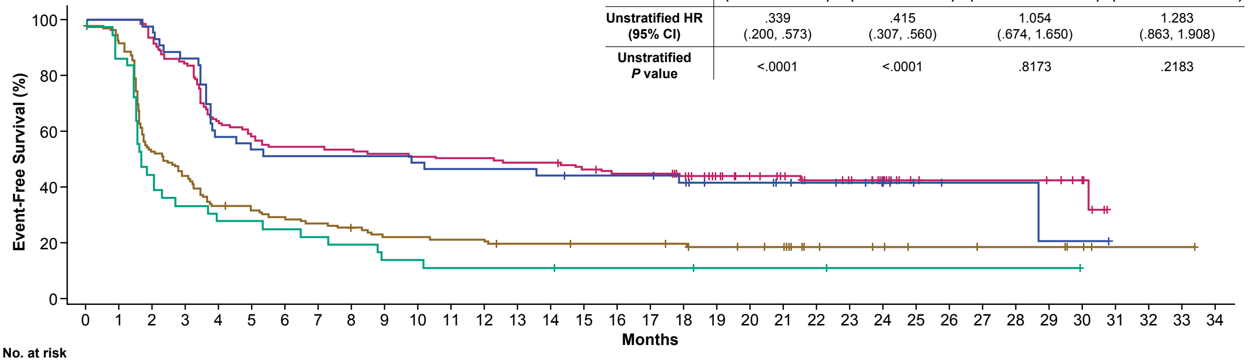

	LDH >2×ULN (Axi-Cel vs SOC)	LDH ≤2×ULN (Axi-Cel vs SOC)	Axi-Cel LDH (>2×ULN vs ≤2×ULN)	LDH >2×ULN (>2×ULN vs ≤2×ULN)
**Unstratified HR (95% CI)**	.339 (.200, .573)	.415 (.307, .560)	1.054 (.674, 1.650)	1.283 (.863, 1.908)
**Unstratified *P* value**	<.0001	<.0001	.8173	.2183

No. at risk

Months	0	1	2	3	4	5	6	7	8	9	10	11	12	13	14	15	16	17	18	19	20	21	22	23	24	25	26	27	28	29	30	31	32	33	34
Axi-Cel, LDH >2xULN	43	43	42	37	25	23	22	22	22	22	21	20	20	20	19	18	18	18	16	13	13	11	10	9	6	3	2	2	2	1	1	0			
Axi-Cel, LDH ≤2xULN	127	127	119	107	80	74	69	69	68	66	65	64	64	62	62	58	55	55	50	43	38	34	29	27	20	11	10	10	10	9	5	0			
SOC, LDH >2xULN	36	31	16	12	10	10	9	8	7	5	5	4	4	4	4	3	3	3	2	2	2	2	1	1	1	1	1	1	1	1	0				
SOC, LDH ≤2xULN	132	118	68	57	43	40	36	34	31	27	27	26	25	23	23	22	22	22	21	19	18	16	10	9	8	6	6	5	5	5	3	1	1	1	0

c **Top Quartile SPD Patients (ZUMA-7)**

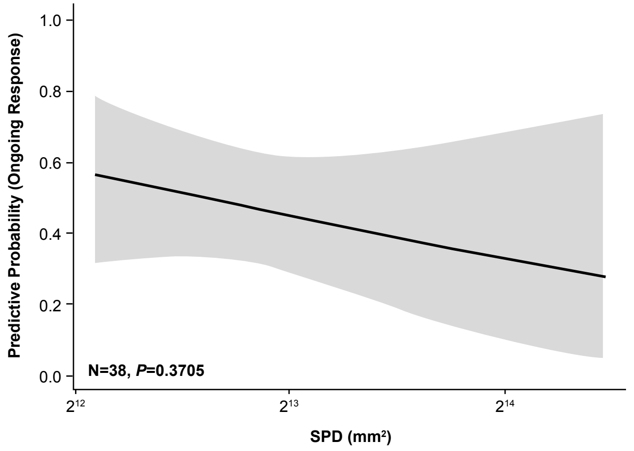

d **>Median SPD Patients (ZUMA-7)**

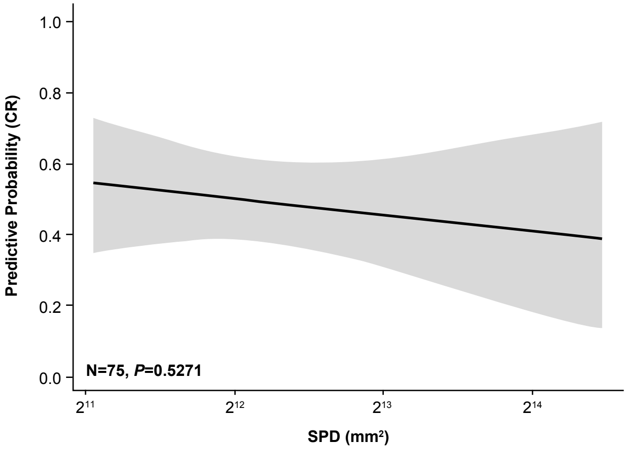

Supplementary Figure 7. Principal Component Analysis of Gene Expression Derived Features Significantly Associated with Clinical Outcome

Principal Component Analysis (PCA) of all genomic features derived from Nanostring expression profiling which were significantly associated with clinical efficacy in the Axi-cel or SOC arm (p-value < 0.05; Ongoing Response, EFS, or DOR). Subjects included in this analysis were those with Nanostring expression profiling of pretreatment tumor biopsies which passed QC (N = 256). (a) PCA loadings plot where the gray arrows represent the Pearson correlation between the listed variable and the two principal components plotted. (b-ad) PCA plots where patients are represented as dots and their location on the plot corresponds to the first two principal component values for each subject. (b-f) Categorical information related to the patient's treatment arm, subtype of disease, or ongoing response status was overlayed on PCA patient plots with category colors indicated in the figure legends. Subjects without data for the corresponding feature are shown in gray. (g-z) Continuous variables derived from Nanostring expression profiling such as Cluster scores, IO360 signature scores, and single gene expression, were log10 transformed and dot colors reflect the subject's value for that feature relative to the median value across all subjects included; gray represents the median value, blue represents values below the median, and red represents values above the median. (aa-ad) Continuous variables which were not derived from Nanostring expression profiling and were associated with efficacy in either treatment arm were log10 transformed and dot colors reflect the subject's value for that feature relative to the median value across all subjects included; gray represents the median value, blue represents values below the median, and red represents values above the median.

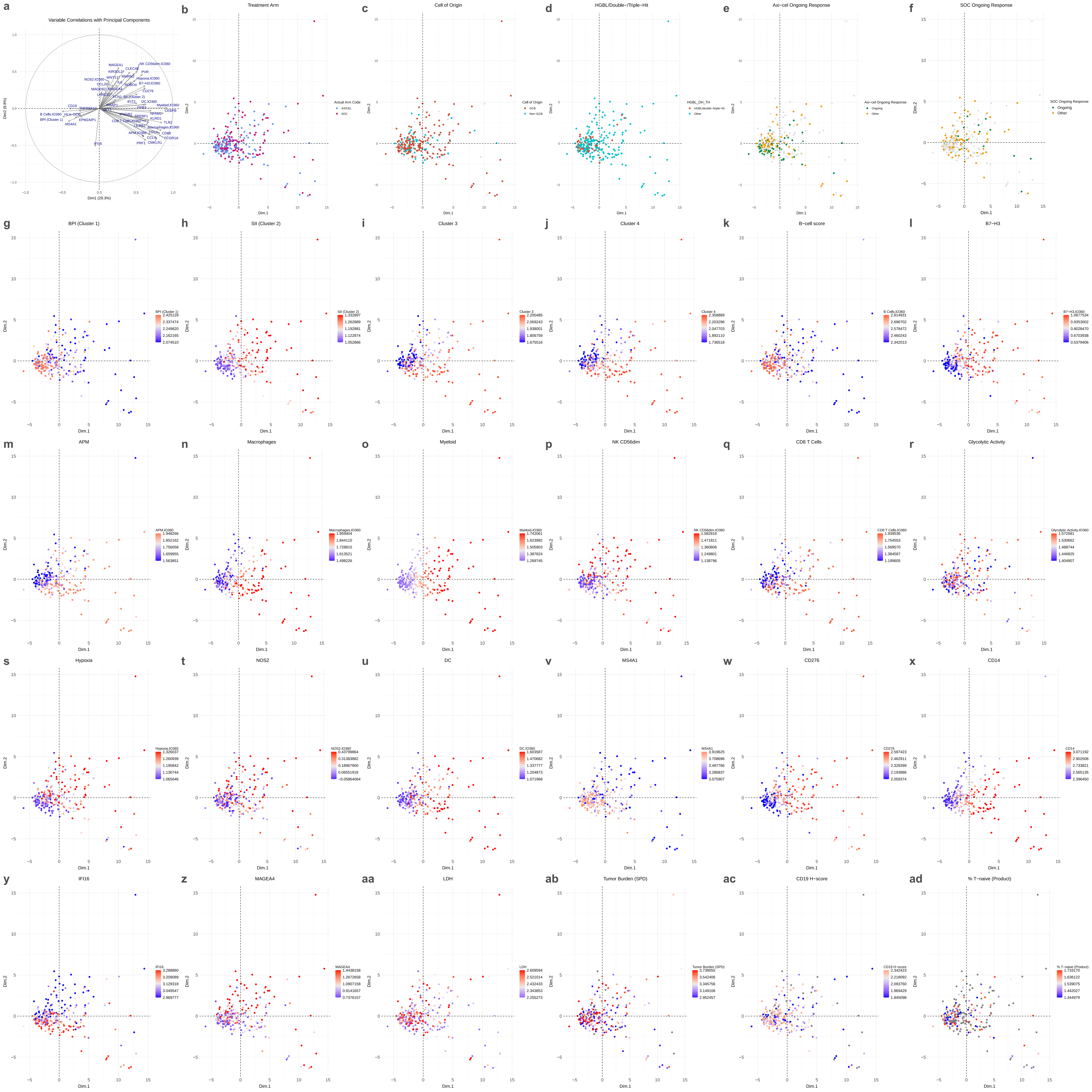

a
Variable Correlations with Principal Components
Dim1 (29.3%)
Dim2 (8.8%)
b
Treatment Arm
Actual Arm Code
AXICEL
SOC
c
Cell of Origin
GCB
Non−GCB
d
HGBL/Double−/Triple−Hit
HGBL_DH_TH
Other
e
Axi−cel Ongoing Response
Ongoing
Other
f
SOC Ongoing Response
Ongoing
Other
g
BPI (Cluster 1)
h
SII (Cluster 2)
i
Cluster 3
j
Cluster 4
k
B−cell score
l
B7−H3
m
APM
n
Macrophages
o
Myeloid
p
NK CD56dim
q
CD8 T Cells
r
Glycolytic Activity
s
Hypoxia
t
NOS2
u
DC
v
MS4A1
w
CD276
x
CD14
y
IFI16
z
MAGEA4
aa
LDH
ab
Tumor Burden (SPD)
ac
CD19 H−score
ad
% T−naive (Product)
Dim.1
Dim.2
