# Supplementary material for: Impact of tumor microenvironment on efficacy of anti-CD19 CAR T cell therapy or chemotherapy and transplant in large B cell lymphoma
Source: Nat Med. 2024 Jan 17;30(2):507–18. doi: 10.1038/s41591-023-02754-1 (PMC10878966; doi:10.1038/s41591-023-02754-1)
Supplement: Supplementary file 2 — Reporting Summary [file 41591_2023_2754_MOESM2_ESM.pdf]

nature portfolio

Corresponding author(s): Frederick L. Locke, MD

Last updated by author(s): Nov 28, 2023

Reporting Summary

Nature Portfolio wishes to improve the reproducibility of the work that we publish. This form provides structure for consistency and transparency in reporting. For further information on Nature Portfolio policies, see our Editorial Policies and the Editorial Policy Checklist.

Statistics

For all statistical analyses, confirm that the following items are present in the figure legend, table legend, main text, or Methods section.

n/a	Confirmed	
☐	☒	The exact sample size (*n*) for each experimental group/condition, given as a discrete number and unit of measurement
☐	☒	A statement on whether measurements were taken from distinct samples or whether the same sample was measured repeatedly
☐	☒	The statistical test(s) used AND whether they are one- or two-sided<br>*Only common tests should be described solely by name; describe more complex techniques in the Methods section.*
☐	☒	A description of all covariates tested
☐	☒	A description of any assumptions or corrections, such as tests of normality and adjustment for multiple comparisons
☐	☒	A full description of the statistical parameters including central tendency (e.g. means) or other basic estimates (e.g. regression coefficient) AND variation (e.g. standard deviation) or associated estimates of uncertainty (e.g. confidence intervals)
☐	☒	For null hypothesis testing, the test statistic (e.g. *F*, *t*, *r*) with confidence intervals, effect sizes, degrees of freedom and *P* value noted<br>*Give P values as exact values whenever suitable.*
☒	☐	For Bayesian analysis, information on the choice of priors and Markov chain Monte Carlo settings
☒	☐	For hierarchical and complex designs, identification of the appropriate level for tests and full reporting of outcomes
☒	☐	Estimates of effect sizes (e.g. Cohen's *d*, Pearson's *r*), indicating how they were calculated

Our web collection on statistics for biologists contains articles on many of the points above.

Software and code

Policy information about availability of computer code

Data collection	The investigator and study staff of each trial site collected samples, as available, from patients. Clinical data from ZUMA-7 were collected using Medidata Rave® from 77 sites worldwide. Between January 25, 2018, and October 4, 2019, 359 patients underwent randomization.
Data analysis	Plots were analyzed and/or generated using TIBCO Spotfire version 11.4.3, nsolver Analysis software version 4.0, nCounter Advanced Analysis version 2.0.143, an Aperio AT2 slide scanner, SAS version 8.3, R version 4.2.3, or GraphPad Prism version 8.

For manuscripts utilizing custom algorithms or software that are central to the research but not yet described in published literature, software must be made available to editors and reviewers. We strongly encourage code deposition in a community repository (e.g. GitHub). See the Nature Portfolio guidelines for submitting code & software for further information.

Data

Policy information about availability of data

All manuscripts must include a data availability statement. This statement should provide the following information, where applicable:

- Accession codes, unique identifiers, or web links for publicly available datasets
- A description of any restrictions on data availability
- For clinical datasets or third party data, please ensure that the statement adheres to our policy

Kite is committed to sharing clinical trial data with external medical experts and scientific researchers in the interest of advancing public health. As such, Kite shares anonymized individual patient data (IPD) upon request or as required by law and/or regulation. Qualified external researchers may request IPD for studies of Kite or

Gilead compounds approved in the United States and the European Union with a marketing authorization date on or after 1 January 2014 and are publicly listed on clinicaltrials.gov or the European Union-Clinical Trials Registry (EU CTR). For studies of newly approved compounds or indication, the IPD will be available for request 6 months after US Food and Drug Administration (FDA) and European Medicines Agency (EMA) approval. Such requests are at Kite's discretion and are dependent on the nature of the request, the merit of the research proposed, availability of the data, and the intended use of the data. If Kite agrees to the release of clinical data for research purposes, the requestor will be required to sign a data sharing agreement to ensure protection of patient confidentiality before the release of any data. Access can be requested by contacting medinfo@kitepharma.com and requests will be addressed within 60 days.

The NanoString data from ZUMA-7 patients discussed in this publication will be deposited in the National Center of Biotechnology Information Gene Expression Omnibus (GEO) and will be accessible through GEO Series. with the following accession number and access code: [PLACEHOLDER NUMBER] and [PLACEHOLDER CODE].

Human research participants

Policy information about studies involving human research participants and Sex and Gender in Research.

Reporting on sex and gender	Subanalyses based on sex or gender were not performed and are therefore not reported herein. Sex of ZUMA-7 patients was reported by the site in the database. Gender was not recorded in the database.
Population characteristics	Covariates were overall uniform among the analysis subgroups. Subanalyses based on patient demographics or population characteristics were not performed and are therefore not reported herein.
Recruitment	Recruitment of patients for the study was done by the investigators at each site.
Ethics oversight	The protocol was approved by the institutional review board or independent ethics committee at each study site and was provided to the key sponsor contact.

Note that full information on the approval of the study protocol must also be provided in the manuscript.

Field-specific reporting

Please select the one below that is the best fit for your research. If you are not sure, read the appropriate sections before making your selection.

☒ Life sciences ☐ Behavioural & social sciences ☐ Ecological, evolutionary & environmental sciences

For a reference copy of the document with all sections, see nature.com/documents/nr-reporting-summary-flat.pdf

Life sciences study design

All studies must disclose on these points even when the disclosure is negative.

Sample size	For this exploratory analysis, no sample size calculations were performed. Evaluable samples from patients in the safety analysis sets of ZUMA-7 (N=170) and ZUMA-1 Cohorts 1+2 (N=101) were included and analyzed.
Data exclusions	No data were excluded from the analyses.
Replication	The data are from two clinical studies. All analyses were reproduced by at least two independent operators and were further analyzed for correctness. There has been no replication of the clinical study. There are not any additional studies in 2L LBCL where to replicate the observations, yet. The findings reported in the study could be confirmed by prospective validation in subsequent trials.
Randomization	Patients in ZUMA-7 were randomized to axi-cel or historical standard of care therapy, as previously described (Locke, et al. New Engl J Med. 2022;386(7):640-654). For these analyses, patients were analyzed based on their randomized treatment. ZUMA-7 is a phase 3 randomized study of axi-cel Vs SOC. Randomization (allocation factors) were response to first-line therapy (primary refractory, vs relapse ≤ 6 months of first-line therapy vs relapse > 6 and ≤ 12 months of first-line therapy) and second-line age-adjusted International Prognostic Index (IPI) (0 to 1 vs 2 to 3) as assessed at the time of screening. For the exploratory analyses presented in here no further pre-defined allocation of patients was performed and no-covariate control was performed. The analysis grouping was based on treatment arm, axi-cel or SOC, median biomarker values (> median Vs <= median) and outcome, Ongoing response Vs others (by data cut off), as indicated throughout the manuscript.
Blinding	As the ZUMA-7 trial previously underwent unblinding, blinding was not applicable for this exploratory analysis utilizing the ZUMA-7 dataset. Albeit pre-defined in the protocol, these were exploratory analyses, performed after the study outcome had already been unblinded.

Reporting for specific materials, systems and methods

We require information from authors about some types of materials, experimental systems and methods used in many studies. Here, indicate whether each material, system or method listed is relevant to your study. If you are not sure if a list item applies to your research, read the appropriate section before selecting a response.

Materials & experimental systems

n/a	Involved in the study
☒	☐ Antibodies
☒	☐ Eukaryotic cell lines
☒	☐ Palaeontology and archaeology
☒	☐ Animals and other organisms
☐	☒ Clinical data
☒	☐ Dual use research of concern

Methods

n/a	Involved in the study
☒	☐ ChIP-seq
☐	☒ Flow cytometry
☒	☐ MRI-based neuroimaging

Clinical data

Policy information about clinical studies

All manuscripts should comply with the ICMJE guidelines for publication of clinical research and a completed CONSORT checklist must be included with all submissions.

Clinical trial registration
ClinicalTrials.gov: NCT03391466 for ZUMA-7; NCT02348216 for ZUMA-1 Cohorts 1+2

Study protocol
The ZUMA-7 study protocol was published in Locke, et al. New Engl J Med. 2022;386(7):640-654. The ZUMA-1 protocol was published in Neelapu, et al. New Engl J Med. 2017;377(26):2531-2544.

Data collection
Clinical data from ZUMA-7 were collected using Medidata Rave® from 77 sites worldwide. Between January 25, 2018, and October 4, 2019, 359 patients underwent randomization for ZUMA-7. Evaluable samples from patients in the safety analysis sets ofZUMA-7 (N=170) and ZUMA-1 Cohorts 1+2 (N=lOl) were analyzed. For this reason, the number of patients included in each analysis varies based on data availability; for clarity, the specific n values are included within each figure.

Outcomes
Outcomes were not assessed prospectively in this exploratory analysis. The ZUMA-7 dataset was used to uncover novel tumor biomarkers associated with outcome (event-free survival, duration of response, ongoing response, complete response, objective response) to axi-cel or standard of care. ZUMA-7 efficacy endpoints (ORR, best response, EFS, DOR, and ongoing response) utilized the primary analysis data cutoff date. EFS was defined as time from randomization to the earliest date of disease progression per Lugano Classification, commencement of new lymphoma therapy, or death from any cause. Ongoing response was defined as patients who were in ongoing response (CR or partial response [PR]) by the ZUMA-7 primary analysis data cutoff date. Progression after response was defined as patients who achieved a CR or PR and subsequently experienced disease progression. Patients who achieved stable disease or progressive disease as best response were included within the category of no response.

Flow Cytometry

Plots

Confirm that:

☒ The axis labels state the marker and fluorochrome used (e.g. CD4-FITC).

☒ The axis scales are clearly visible. Include numbers along axes only for bottom left plot of group (a 'group' is an analysis of identical markers).

☒ All plots are contour plots with outliers or pseudocolor plots.

☒ A numerical value for number of cells or percentage (with statistics) is provided.

Methodology

Sample preparation
CAR T cell products were manufactured from patient apheresis material. After manufacturing, CAR T cell products were harvested and viably cryo-preserved into small aliquots for subsequent testing. Flow cytometry testing was performed immediately after thawing of the CAR T cell product vials.

Instrument
BD FACSCanto™ II (serial number: R33896203053, reference number: 338962)

Software
Software used for acquisition/collection is BD FACSDiva™ v9.0 and for analysis is FlowJo v10.7

Cell population abundance
The preparation consisted of almost entirely (>90%) T cells because of the manufacturing process utilized to produce axi-cel. Purity is accessed by flow assay looking at viability, CD3+, and CAR+ by QC.

Gating strategy
FSC/SSC gating is used to identify single mononuclear cell population (lymphocytes). To identify single cells, FSC-H/FSC-A gating is used. SSC-A/FSC-A gate identifies total lymphocyte population. Live cells are identified by the viability marker 7-AAD, where live cells are 7-AAD negative. Total viable T cells are identified by positive CD3 staining. Further phenotyping of CD3+ T cells is done by staining with CD4, CCR7, and CD45RA markers. CD8+ T cells are defined as CD3+ and CD4-. For CCR7 and CD45RA, we use FMO (Fluorescence minus one) controls to accurately identify the positive and negative populations for those phenotypes. T cell Memory phenotypes are defined as the following, Naïve T cells (CCR7+ CD45RA+), Central Memory T cells (CD3+ CCR7+CD45RA-), Effector T cells (CD3+ CCR7- CD45RA+), and Effector Memory T cells (CD3+ CCR7- CD45RA-).

☒ Tick this box to confirm that a figure exemplifying the gating strategy is provided in the Supplementary Information.
